# Manipulating Gibberellin Control Over Growth and Fertility as a Possible Target for Managing Wild Radish Weed Populations in Cropping Systems

**DOI:** 10.3389/fpls.2020.00190

**Published:** 2020-03-19

**Authors:** Michael Groszmann, Peter M. Chandler, John J. Ross, Steve M. Swain

**Affiliations:** ^1^Division of Plant Sciences, Research School of Biology, Australian National University, Canberra, ACT, Australia; ^2^CSIRO Agriculture and Food, Canberra, ACT, Australia; ^3^School of Biological Sciences, University of Tasmania, Hobart, TAS, Australia

**Keywords:** weed control, overgrowth mutants, gibberellin 3-oxidase, *Raphanus raphanistrum*, plant fertility, GID1-Della yeast two-hybrid system, plant growth regulator (PGRs), seed set efficiency

## Abstract

Wild radish is a major weed of Australian cereal crops. A rapid establishment, fast growth, and abundant seed production are fundamental to its success as an invasive species. Wild radish has developed resistance to a number of commonly used herbicides increasing the problem. New innovative approaches are needed to control wild radish populations. Here we explore the possibility of pursuing gibberellin (GA) biosynthesis as a novel molecular target for controlling wild radish, and in doing so contribute new insights into GA biology. By characterizing *ga 3-oxidase* (*ga3ox*) mutants in *Arabidopsis*, a close taxonomic relative to wild radish, we showed that even mild GA deficiencies cause considerable reductions in growth and fecundity. This includes an explicit requirement for GA biosynthesis in successful female fertility. Similar defects were reproducible in wild radish *via* chemical inhibition of GA biosynthesis, confirming GA action as a possible new target for controlling wild radish populations. Two possible targeting approaches are considered; the first would involve developing a species-specific inhibitor that selectively inhibits GA production in wild radish over cereal crops. The second, involves making crop species insensitive to GA repression, allowing the use of existing broad spectrum GA inhibitors to control wild radish populations. Toward the first concept, we cloned and characterized two wild radish *GA3OX* genes, identifying protein differences that appear sufficient for selective inhibition of dicot over monocot GA3OX activity. We developed a novel yeast-based approach to assay GA3OX activity as part of the molecular characterization, which could be useful for future screening of inhibitory compounds. For the second approach, we demonstrated that a subset of GA associated *sln1*/*Rht-1 overgrowth* mutants, recently generated in cereals, are insensitive to GA reductions brought on by the general GA biosynthesis inhibitor, paclobutrazol. The location of these mutations within sln1/*Rht-1*, offers additional insight into the functional domains of these important GA signaling proteins. Our early assessment suggests that targeting the GA pathway could be a viable inclusion into wild radish management programs that warrants further investigation. In drawing this conclusion, we provided new insights into GA regulated reproductive development and molecular characteristics of GA metabolic and signaling proteins.

## Introduction

### Wild Radish Weed Biology

The cruciferous plant wild radish (*Raphanus raphanistrum*) is a native to Mediterranean regions, but has established itself as formidable broad leaf weed species of cereal crops in North America and Australia (Piggin et al., 1978). Wild radish is a significant problem for the Australian wheat industry being the most economically damaging dicotyledonous weed species in Australian cropping systems. It accounts for an estimated $53–72 million per year in crop losses (Jones et al., 2005; Llewellyn et al., 2016) with this figure in danger of increasing with the continued emergence of herbicide resistant populations (Hashem et al., 2001; Walsh et al., 2004; Walsh et al., 2007; Ashworth et al., 2014). The development of new control methods include, research into novel molecular targets (Newbigin et al., 2006; Swain et al., 2006; Young et al., 2008) that can be combined with better farming practices, as an integrated management method for combating wild radish (Cheam and Cheam, 2008; Walsh et al., 2013; Walsh et al., 2017; Walsh, 2018).

Wild radish has a unique biology that makes it hugely successful as a weed. Its rapid establishment and fast growth rate ensures it outcompetes neighboring crop plants for nutrients, light, and soil moisture. Wild radish also produces copious seed numbers with over 1,000 seeds/plant and >45,000 seed/m^2^ with dense infestations (Reeves et al., 1981; Blackshaw et al., 2002; Eslami et al., 2006). Its complex seed dormancy mechanisms allow for germination at opportune times during a season and over several years. As an outcrossing species, wild radish is proficient at sharing and spreading genetic change, which has led to the spread and increased prevalence of herbicide resistance across key cereal growing regions of Australia (Owen et al., 2015). Therefore slowing growth rates, and more importantly reducing fertility, are key focal points when considering new molecular approaches for controlling wild radish populations.

### Gibberellins Control Plant Growth and Fertility

Growth and developmental processes are largely regulated by plant hormones, such as gibberellin (GA) (Davies, 2012). GAs are involved in a diverse range of growth and developmental processes throughout the life cycle of a plant and constitute a large group of diterpenoid compounds, generally named in order of discover GA_1-n_. Most GA molecules are inactive precursors or de-activated catabolites of the biologically active GAs, the most common bioactive variants being GA_4_ and GA_1_ (Thomas and Sun, 2004; Yamaguchi, 2008; Claeys et al., 2014). GA biosynthesis involves a progressive series of oxidation and hydroxylation reactions beginning with modification of geranylgeranyl diphosphate (GGDP) in the plastid, moving to the ER, and concluding in the cytosol with the production of immediate precursors GA_9_ or GA_20_ by GA20-OXIDASE (GA20OX) and conversion into bioactive GA_4_ or GA_1_, respectively, by GA3-OXIDASE (GA3OX) (Yamaguchi, 2008). The extended metabolic pathway offers multiple regulatory points to control endogenous GA levels, which are further maintained by inactivation of bioactive GA by yet further hydroxylation modifications catalyzed by GA2-OXIDASE (GA2OX) (Yamaguchi, 2008). Bioactive GAs are categorized as such because of their ability to interact with the GA receptor (*GID1*) to initiate interaction and targeted degradation of DELLA proteins that repress growth and developmental processes (Ueguchi-Tanaka et al., 2007; Sun, 2011; Claeys et al., 2014; Van De Velde et al., 2017b).

The genes encoding the essential proteins for GA production, degradation, perception, and signaling have all been identified in multiple species (Hedden and Thomas, 2012). Many mutants deficient in either production or perception of GA have been well characterized in the model species *Arabidopsis*, a member of the Brassicaceae and thus a close taxonomic relative to wild radish. These mutants show a range of developmental abnormalities and severities depending on the degree to which GA production/signaling is impaired. Defects often include reduced germination rates, delayed seedling establishment, smaller overall size, delayed flowering time, and floral defects affecting reproductive organs and consequently a reduced seed set or even total sterility (Hedden and Thomas, 2012).

In addition to mutant approaches, reductions in the GA content of plants can be achieved through application of plant growth regulators that interfere with GA production. These chemicals have been frequently used in agriculture and horticulture since the 1950s to manipulate plant growth to desired requirements (Rademacher, 1991; Rademacher, 2018). GA inhibiting chemicals are grouped based on composition and targeted enzyme (Rademacher, 2000; Rademacher, 2018). The “onium” compounds (e.g., chlormequat chloride and AMO-1618) and nitrogen-containing heterocycle compounds (e.g., paclobutrazol and ancymidol) block GA biosynthesis early in the pathway. Prohexadione-calcium and other group three members are structurally similar to the co-substrate of the 2-oxoglutarate dependent dioxygenase enzymes (e.g., GA20-OXIDASE and GA3-OXIDASE) and thus interfere with the final steps of GA biosynthesis. The 16,17-dihydro-GAs are modified derivatives of active GA precursors that function as competitive inhibitors, interfering with the later GA biosynthesis enzymes such as GA3OX (Rademacher, 2018). Interestingly, the 16,17-dihydro-GA inhibitors display species discrimination, acting as an effective growth retardant in monocot species but not in dicots (Evans et al., 1994; Foster et al., 1997; King et al., 2004; Rademacher, 2018).

### Targeting Gibberellin Biosynthesis as a Novel Molecular Means of Combating Wild Radish

To determine if reducing GA levels in wild radish would negatively impact its vigorous growth and reproductive capacity, we first examined phenotypes arising from GA-deficiency in the model plant *Arabidopsis*, a close *Brassica* relative of wild radish. Severe GA-deficient mutants in *Arabidopsis* often involve enzymes early in the biosynthesis pathway and are non-germinating, non-flowering, and are extremely dwarfed (Thomas and Sun, 2004). It is improbable that field applications of a chemical GA inhibitor could reliably reproduce such dramatic abnormalities in wild radish. Instead we chose to examine GA-deficient mutants with milder defects reflective of partial suppression of GA levels. We focused on genes encoding for GA3OX proteins as they catalyze the final step of GA_9_ to GA_4_ and are targets of a number of GA inhibitors, including the species-specific 16,17-dihydro-GAs inhibitors. In *Arabidopsis*, four genes encode GA3OX proteins and due to this partial redundancy, a mutation in one or two of these *GA3OX* genes reduces but does not abolish GA_4_ levels (Chiang et al., 1995; Mitchum et al., 2006) and are therefore more comparable to the expected outcome from applying a GA inhibitor.

We show that even mild GA deficiencies cause considerable reductions in growth and specific aspects of fecundity in *Arabidopsis*, which are reproducible in wild radish using a chemical GA inhibitor. We then explore two possible approaches to targeted GA inhibition in the dicot weed over the monocot crop. The first, would involve developing a species-specific inhibitor that selectively targets GA production in wild radish. The second approach would involve making crop species insensitive to GA inhibitors, allowing the use of existing non-species-specific GA inhibitors to control wild radish populations.

### The Potential to Develop a Wild Radish-Specific Gibberellin Inhibitor

Toward exploring the possibility of a wild radish specific GA inhibitor, we cloned and characterized two *GA3OX* genes from wild radish (*RrGA3OX1a* and *RrGA3OX2a*) and identified the other members of the *RrGA3OX* family. As part of the functional characterization of the GA3OX enzymes, we developed a yeast-based system to assay GA 3-oxidase activity that could be useful in future screening of inhibitory compounds. This system is based on previous reports showing that in plants, the interaction between the GA receptor (GID1), and DELLA proteins occur due to a conformational change in GID1 brought on by binding a “biologically active” GA, such as GA_4_ (Ueguchi-Tanaka et al., 2005). This GA_4_ dependent GID1-DELLA protein-protein interaction can be recreated heterologously in yeast using the yeast two-hybrid (Y2H) system and assayed through either inactivation (GA_4_ absent) or activation (GA_4_ present) of reporter genes (Ueguchi-Tanaka et al., 2007). We show that upstream components of the GA biosynthesis system can be engineered into this system allowing inactive precursor GAs to be fed to the yeast, which are subsequently metabolized into bioactive GAs and detectable through the GID1-DELLA Y2H activation of a reporter gene. We next used extensive phylogenetic comparisons, protein sequence analysis, and protein homology modeling, to map regions and residue differences of likely functional importance between GAOX classes and more specifically between dicot and monocot GA3OX proteins. It appears that sufficient differences exist between wheat and wild radish GA3OX proteins to reasonably expect that discriminating GA inhibitors could be developed.

### Engineering Gibberellin Inhibitor Resistant Crops as an Alternative Approach

Instead of developing species-specific GA inhibitors, cereal crops could be engineered that are insensitive to general GA inhibitors. This could be accomplished by elevating the levels of proteins targeted by GA biosynthesis inhibitors such as paclobutrazol (Swain et al., 2005). Alternatively, mutants could be used that uncouple plant growth from GA signaling. This would involve altering the activity of the growth suppressing DELLA proteins which are targeted for degradation as part of the early events associated with GA signaling (Sun, 2011). At one extreme, of uncoupling plant growth from GA signaling, are mutants with a complete loss of *DELLA* function. These “slender” mutants have excessive elongated growth through a state analogous to high GA levels and constitutive GA signaling. At the other end of the spectrum are mutant *DELLA* proteins that are resistant to GA-mediated degradation. These GA-insensitive *della* mutants act as if in a perpetual GA depleted state and are unresponsive to changes in GA levels due to a reduced affinity for the GA receptor (Feng et al., 2008). Their effects on growth depends on the level of resistance that a given mutant della protein has to GA-mediated degradation, and the redundancy with other *DELLA* genes present in the genome. Mutants of both extremes of GA signaling have been characterized in a range of species including crops such as rice, maize, wheat, and barley. The “slender” mutants are of no agricultural use given their weak spindly growth and reduced fertility (Lanahan and Ho, 1988; Dill and Sun, 2001; Ikeda et al., 2001; Chandler et al., 2002; Kay et al., 2013; Plackett et al., 2014). Whereas, mild semi-dwarfing “GA-insensitive” alleles have been widely used in modern agriculture for their lodging resistance and spectacular harvest index since the green revolution of the 1960s (Peng et al., 1999; Hedden, 2003). Theoretically, a strong “GA-insensitive” *DELLA* mutant would confer the best resistance to general GA inhibitors given their indifference to GA levels. However, such alleles would be excessively detrimental to plant growth with even moderately strong alleles in barley (*sln1d*) and wheat (*Rht-B1c*) being too dwarfed for agricultural purposes (Chandler and Harding, 2013; Derkx et al., 2017). Therefore the “ideal” mutant would be a DELLA protein that is largely insensitive to GA levels, with a diminished growth-suppressing activity, that offsets growth reductions to within a more acceptable range.

Recently a new set of *SLN1* (barely) and *RHT-B1* (wheat) *DELLA* alleles (termed “*overgrowth*” mutants) have been identified in a suppressor screen of the strong “GA-insensitive” overly dwarfed *sln1d* and *Rht-B1c* mutants. These second site mutations result in taller plants closer to the semi-dwarf ideotype (Chandler and Harding, 2013) and may embody our proposed ideal mutant (i.e., still GA insensitive but with less growth suppression). These suppressing alleles were found to be secondary point-mutations within *SLN1* and *RHT-B1* itself, which likely impairs the capacity of the DELLA protein to execute downstream growth-suppressing functions (Chandler and Harding, 2013; Van De Velde et al., 2017b). This accounts for the increased growth of the suppressor alleles over their *sln1d* and *Rht-B1c* progenitors, but also means the intrinsic insensitivity to GA levels due to the original *sln1d* and *Rht-B1c* mutation may remain.

We show that a subset of these GA associated *sln1*/*rht-B1 overgrowth* mutant suppressor alleles in cereals, are insensitive to reductions in GA levels brought on by the broad spectrum GA biosynthesis inhibitor, paclobutrazol. In addition, the location of these mutations within *SLN1*/*RHT-B1* provides additional insight into the functional domains of these important GA signaling proteins and extends the potential applications of these *overgrowth* alleles currently being integrated into cereal breeding programs.

## Results

### Reduced Gibberellin Levels Cause Dwarfism and Reduced Fertility in *Arabidopsis*, a Close *brassica* Relative of Wild Radish

**Figure 1:**
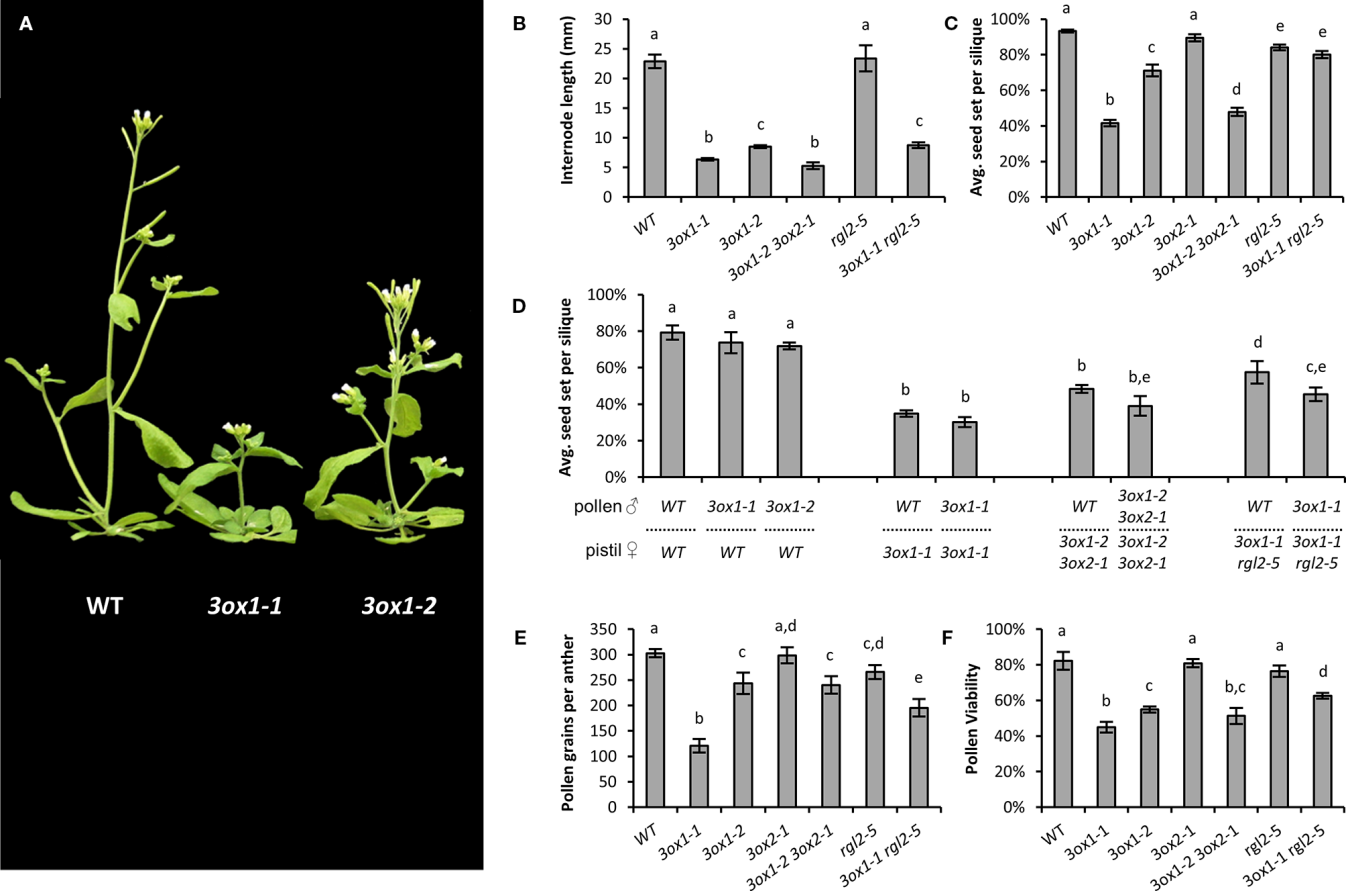
Growth and fertility defects of Arabidopsis *ga3ox* mutants. **(A)** Representative plants depicting the differences in plant stature between wild type (WT), the stronger *ga3ox1-1*, and intermediate *ga3ox1-2* mutants. **(B)** Effects on plant height reported as average internode length. **(C)** Fecundity of the various mutant lines. Seed set per silique = seed/(seed + unfertilized ovules). **(D)** Reciprocal hand pollinations showing that the reduced fecundity of *ga3ox* mutants involve both male and female defects. **(E, F)** Phenotypic defects associated with pollen. *ga3ox1* mutants show reduced pollen production **(E)**, with the pollen that is produced having reduced viability **(F)**. Letters above bars represent pairwise statistical comparison between categories. Categories marked with a given letter are statistically different from categories marked with another letter (Student’s *t*-test p < 0.05). All error bars are S.E.M. N = **(B)** 20–40 plants; **(C)** 30–45 siliques from across 4–5 per plants; **(D)** 10–25 crosses; **(E)** 10–15 anthers, each from a unique flower; **(F)** pollen from 8 to 10 anthers, each from a unique flower.

*GA3OX1* is the most ubiquitously expressed of the four *GA3OX* genes in *Arabidopsis* (Mitchum et al., 2006). Compared to wild type (WT) plants, *3ox1* mutants are slow growing and semi-dwarfed, with the missense *3ox1-1* mutant allele being more severe than the T-DNA null *3ox1-2* allele (**Figures 1A, B**). The *3ox1-1* allele carries a point mutation changing a conserved cysteine residue that severely reduces catalytic activity (Chiang et al., 1995). Through a “low-GA” feed-back mechanism, the expression levels of *GA3OX1* increase, presumably elevating protein levels, which in the *3ox1-1* mutant results in a highly expressed protein capable of pre-cursor GA_9_ interaction but with limited conversion to bioactive GA_4_ (Chiang et al., 1995). Substrate squelching and competition with the other three *GA3OX* proteins likely confers the more severe GA deficiency of *3ox1-1* compared to the absence of the GA3OX1 protein in the *3ox1-2* mutant. The *3ox1-1* mutant exhibited a 72% reduction in average internode length, whereas the milder defects of *3ox1-2* were magnified by co-compromising *GA3OX2* function (i.e., *3ox1-2 3ox2-1* double mutant; **Figures 1A, B**). The severely dwarfed stature of both the *3ox1-1* and the *3ox1-2 3ox2-1* mutants could be rescued by exogenous GA application in a dose dependent manner (**Supplementary Figure S1A**), confirming that the diminished growth in these mutants is a consequence of reduced GA levels. We also tested the connection with GA genetically by using a mutant of *RGL2* (*RGA-LIKE 2*). *RGL2* encodes for one of the five DELLA proteins that repress GA-regulated growth and development responses, and are degraded through the action of bioactive GA. *RGL2* is only weakly expressed in vegetative tissues, but expressed highly in floral organs where it controls development of reproductive tissues (Klepikova et al., 2016; Gómez et al., 2019). Despite weak vegetative expression, a loss-of-function *rgl2* should still partially alleviate low-GA induced growth suppression. Consistent with a GA deficiency, the stature of *3ox1-1* was slightly improved by co-loss of RGL2 function as seen in the *3ox1-1 rgl2-5* double mutant (**Figure 1B**).

Compromising *GA3OX* activity also reduced seed set to varying degrees between the *3ox* mutant alleles (**Figure 1C**), correlating with the severity of the mutant alleles on vegetative development. The greatest reduction occurred in the *3ox1-1* and *3ox1-2 3ox2-1* mutants, with seed yields below half that of WT (**Figure 1C**). The poor seed set of *3ox1-1* was almost completely rescued in the *3ox1-1 rgl2-5* double mutant (**Figure 1C**) and could be improved upon application of GA (**Supplementary Figure S1B**); confirming that the reduced fertility is related to decreased GA production.

We found that the diminished seed set of *3ox* mutants involves limitations in both male and female fertility. The decrease in female fertility was evident by a consistent reduction in the fertilization rate of ovules, to produce seed, in all *3ox* mutant combinations (i.e., *3ox1-1*, *3ox1-2 3ox2-1*, and *3ox1-1 rgl2-5*), even when pollinating with WT as the male donor (**Figure 1D**). There were no obvious signs of post-fertilization seed abortion, but clear signs of unfertilized ovule senescence, indicating the reduced seed set was due to a failure in ovule fertilization. The deficiencies in *3ox* male fertility begins with malformed anthers that produce fewer pollen grains compared to WT (**Figure 1E**; **Supplementary Figure S1C**). The pollen that is produced by the *3ox* mutants is less viable with impaired germination efficiency than WT (**Figure 1F**; **Supplementary Figure S1D**). The addition of GA to the pollen germination medium failed to improve *3ox1-1* or *3ox1-2* pollen germination rates (data not shown). A concurrent loss of RGL2 function in the *3ox1-1 rgl2-5* double mutant was able to improve pollen germination, viability, and production over the *3ox1-1* single mutant (**Figures 1E, F**; **Supplementary Figure S1D**). The effect on fecundity of these pollen defects is observed as a consistent trend of reduced seed set when applying *3ox* mutant pollen compared to WT pollen, irrespective of the female genotype (**Figure 1D**). The impact of these *3ox* associated male fertility defects on seed set are likely to be underestimated given the disproportionately larger ratio of pollen grains deposited on the stigma (hundreds) to ovules within the pistil (~50) being a likely alleviating factor.

The *Arabidopsis* results are promising indications that decreasing GA levels will detrimentally impact key traits making wild radish a successful weed species. The anther/pollen defects could possibly be more impactful in wild radish which is an obligate out-crossing species where pollen deposits on a distant recipient stigma will be considerably less than in the self-pollinating *Arabidopsis*.

### Reductions in Gibberellin Levels Decreases Wild Radish Growth and Reproduction Rates

**Figure 2:**
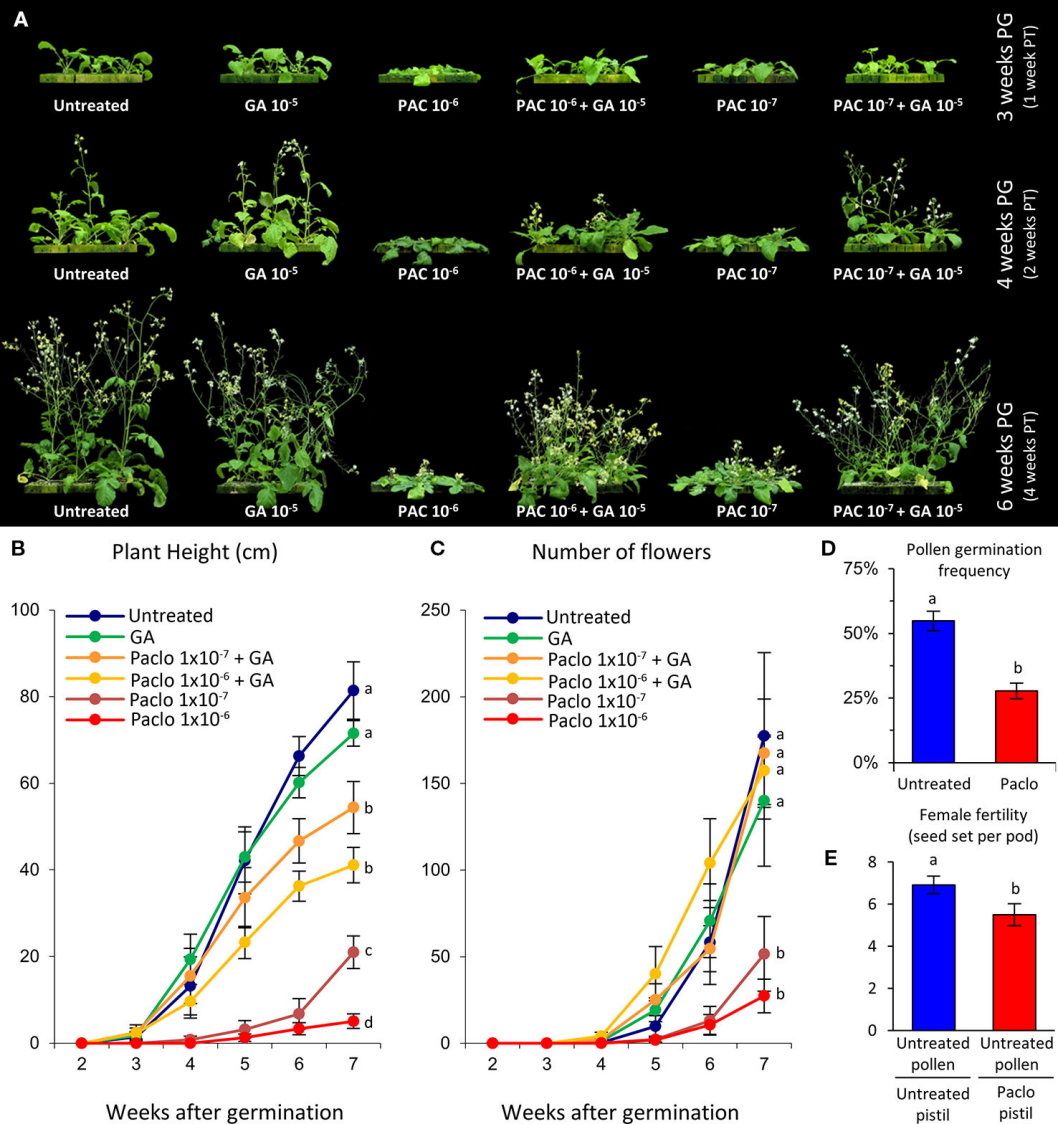
Growth and fertility defects induced by paclobutrazol treatment of hydroponically grown wild radish. **(A)** Representative images of wild radish showing the impact on growth induced by paclobutrazol (Paclo) treatment. PG, post-germination; PT, post-treatment. **(B)** Growth, represented as plant height, over a 7 week period. Lettering denotes statistical differences between categories at week 7. **(C)** Average number of flowers (reproductive units) produced per plant across the different treatments. Lettering denotes statistical differences between categories at week 7. **(D)** *In vitro* pollen germination frequency showing significantly reduced viability of pollen produced by paclobutrazol treated wild radish plants. **(E)** Reduction in female fertility seen in paclobutrazol treated plants. Pollen (male) donor from PG4 population [mate compatible with Albany (AL) population]. Pistil (female) recipient from untreated and treated AL wild radish plants. Lettering adjacent to data points in panels **(B–E)** denote pairwise statistical comparison between categories. Categories marked with a given letter are statistically different from categories marked with another letter (Student’s *t*-test p < 0.05). All error bars are S.E.M. N = **(B**, **C)** 10 plants for each treatment; **(D)** 10 anthers, 1 anther from 10 independent flowers; **(E)** 20 siliques.

The commercially available GA biosynthesis inhibitor paclobutrazol was used to evaluate the effects of reducing GA levels on wild radish development (**Figure 2**). Wild radish plants were grown for 2 weeks in GroWool and then subjected to a single dose of either paclobutrazol and/or GA_3_ (see *Experimental Procedures*). At 4 weeks post-germination (2 weeks after treatment), plants from the nutrient-only control (untreated) and GA-only groups were comparable in size and development, producing large rosettes with broad long leaves and had transitioned to flowering with clearly elongated main shoots (**Figures 2A, B**). In contrast, wild radish plants treated with paclobutrazol were dwarfed with compact rosettes, consisting of smaller and darker green leaves, and although they had transitioned to flowering the elongation of the bolt was minimal (**Figures 2A, B**). Rosette development of plants simultaneously treated with paclobutrazol and GA were, in a dose dependent manner, substantially increased compared to the paclobutrazol treated plants, being more comparable in size and appearance to the control plants (**Figures 2A, B**). This indicates that the measured phenotypic effects of the paclobutrazol treatment appear predominantly GA related.

By 6 weeks post-germination the development of the paclobutrazol treated wild radish were dramatically different compared to untreated plants. The rosette of the paclobutrazol plants were reduced in size with diminutive plant height (**Figures 2A, B**), and flower production was diminished (**Figure 2C**). These phenotypic defects induced by paclobutrazol were dose dependent (**Figures 2A–C**). Rosette size was comparable between the untreated, GA-only, and paclobutrazol+GA plants indicating that the altered vegetative growth of the paclobutrazol treated plants was a consequence of reduced GA levels. However, main stem height and average internode length of the paclobutrazol+GA plants was only approximately two-thirds that of the control and GA-only groups (**Figure 2B**). This lack of complete rescue could reflect non-GA-specific paclobutrazol effects on shoot elongation, or alternatively sub-optimal availability of exogenous GA (applied as root drench) to the developing shoot. The latter would be consistent with not obtaining taller plants in the GA-only group compared to the control group, despite the known general positive influence of GA on internode elongation (Yamaguchi, 2008). To examine this further, main shoots from an independent sowing of wild radish plants were excised and placed into nutrient solution containing paclobutrazol and/or GA and allowed to grow for 2 weeks. In these cuttings, GA enhanced stem growth by ~40%, while paclobutrazol reduced stem elongation by ~50%. Co-treatment with paclobutrazol and GA improved stem elongation over paclobutrazol-only by ~150%, restoring stem elongation to levels equivalent to the untreated group and approaching the GA-only group (**Supplementary Figure S2A**). In addition, GA stimulated parthenocarpic fruit growth was observed in the GA and paclobutrazol+GA group, while pistils on paclobutrazol treated cuttings were reduced in length (**Supplementary Figure S2B**). These results indicate that the reductions in plant height observed in the growth assay, depicted in **Figure 2**, are most likely a consequence of reduced GA levels; with the inability of our GA application to fully restore paclobutrazol treated plant height most likely due to sub-optimal availability of the GA to the stem tissue *via* the root drench application.

By 7–8 weeks post-germination the paclobutrazol treated plants showed a 60–80% reduction in flower number compared to untreated plants (**Figure 2C**), which was rescued by co-application of GA (**Figure 2C**). As seen in the *Arabidopsis ga3ox* mutants, the anthers from the paclobutrazol treated wild radish were often malformed and lacking the copious pollen production of untreated plants. The pollen that was produced, germinated *in vitro* at only half the frequency observed for the control group (**Figure 2D**). The pistils were slightly shorter (**Supplementary Figure S2B**) and seed set was reduced by ~26%, even with application of copious pollen from untreated fertilization compatible (PG4 population) plants (**Figure 2E**). These results are consistent with our observations of fertility defects in *Arabidopsis ga3ox* mutants, including a female-specific reduction in fertility.

A second experiment evaluating the effects of reducing GA levels on wild radish development was conducted, this time using soil as the growth medium. As seen with the first growth experiment, paclobutrazol severely limited the vegetative and reproductive growth of wild radish; effects that could be generally rescued by co-application of GA (**Supplementary Figure S3**).

The phenotypes of the paclobutrazol treated wild radish, resemble the aberrant development of *Arabidopsis ga3ox* mutants, supporting that these observed defects in wild radish are a consequence of reduced GA levels. Collectively, these observations reveal firstly, that GA is required for normal growth and fecundity of wild radish and secondly, that reducing GA levels impacts negatively on traits important for its success as a weed.

### Cloning and Identification of Gibberellin 3-OXIDASE Genes From Wild Radish

**Figure 3:**
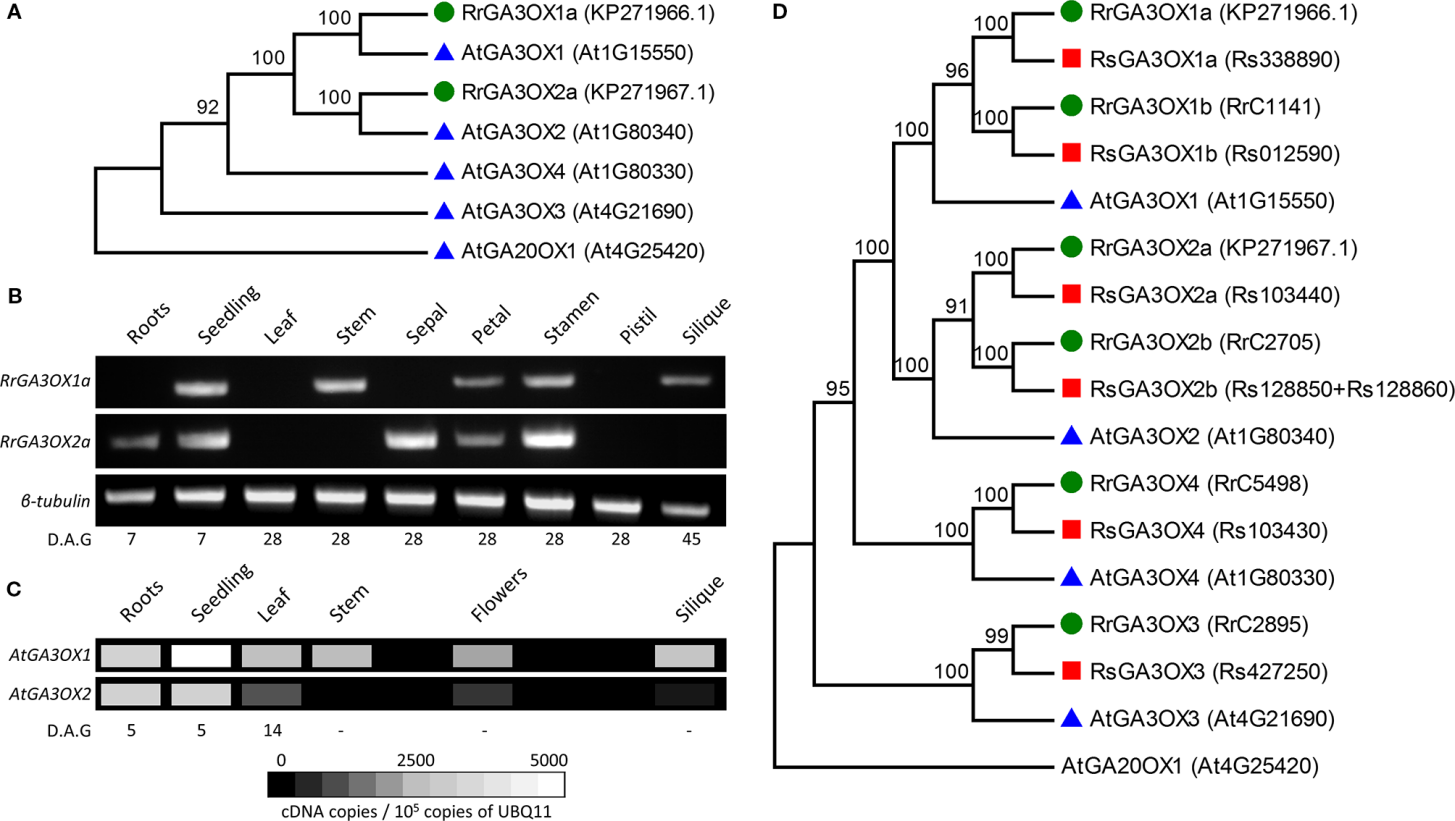
Characterization of wild radish *GA3OX* genes. **(A)** Phylogenetic relationship between the protein sequence of the two cloned wild radish GA3OX genes (*RrGA3OX1a* and *RrGA3OX2a*; green circles) and those of *Arabidopsis* (blue triangles). *AtGA20OX1* represents an out-group. GenBank (*Rr*) and TAIR (*At*) gene/protein identifiers are in parentheses adjacent to gene names. **(B)** Semi-quantitative real time (RT)-PCR showing *RrGA3OX1a* and *RrGA3OX2a* expression in a range of wild radish tissues. For primer specificity, see **Supplementary Figure S5**. **(C)** Virtual gel heat map showing quantitative expression levels of *AtGA3OX1* and *AtGA3OX2* in different *Arabidopsis* tissues. Data adapted from quantitative PCR (qPCR) analysis published in Mitchum et al., 2006. **(D)** Phylogenetic relationship between *Arabidopsis* (AtGA3OX; blue triangles), wild radish (*Raphanus raphanistrum*; RrGA3OX; green circles), and cultivated radish (*Raphanus sativus*; RsGA3OX; red squares) GA3OX protein sequences. The genomes of both wild radish and *R. sativus* carry six *GA3OX* genes consisting of two ohnologs for both *GA3OX1* and *GA3OX2*, in addition to single copies of *GA3OX3* and *GA3OX4* paralogs. GenBank (*RrGA3OX1a and RrGA3OX2a*), RadishDB (other *Rr*), RadishGB (*Rs*), and TAIR (*At*) gene/protein identifiers are in parentheses adjacent to gene names.

As some GA3OX inhibitors display different effectiveness between species, we focused on examining *GA3OX* genes as possible molecular targets in wild radish. *RrGA3OX* genes expressing in the anthers were a principal target given the considerable importance of pollen production in an obligate out-crossing species. Candidate *RrGA3OX* genes were isolated using degenerate primers designed from a region within *GA3OX* genes conserved across *Arabidopsis* and a number of other Brassicaceae. Two variants of partial complementary DNAs (cDNAs) were identified from wild radish (AL population) anthesis stage anthers and full-length clones were obtained using 5′- and 3′-rapid amplification of cDNA ends (RACE) (**Supplementary Figures S4A, B**). The two genes were designated *RrGA3OX1a* and *RrGA3OX2a* based on homology to the *Arabidopsis* GA3OX proteins (**Figure 3A**), being ~85% homologous to AtGA3OX1 and AtGA3OX2 respectively (**Supplementary Figure S4D**). The suffix “a” was appended to distinguish these specific *RrGA3OX* genes from anticipated ohnologs in the wild radish genome, given its recent hexaploid ancestry (Moghe et al., 2014). Semi-quantitative real-time (RT)-PCR, using primer sets that differentiate between the two *RrGA3OX* variants (**Supplementary Figure S5**), revealed that the two genes are expressed in a range of tissues throughout the plant, in patterns distinct from one another (**Figure 3B**). Both *RrGA3OX1a* and *RrGA3OX2a* co-express in the aerial tissues of young developing seedlings, and in the petals and stamens of the flower. *RrGA3OX1a* expression was uniquely detected in the stem and siliques, while *RrGA3ox2a* was unique in its root and sepal expression. These expression localizations match with tissues all known to require bioactive GA for their proper development (Hedden and Thomas, 2016).

Comparing the reported expression patterns and levels of *AtGA3OX1* and *AtGA3OX2* (Mitchum et al., 2006; Hu et al., 2008) (**Figure 3C**), with those of *RrGA3OX1a* and *RrGA3OX2a*, revealed a degree of conservation in tissue-specificity between the orthologous genes. All four *GA3OX* genes express strongly in the aerial tissues of young developing seedlings (**Figures 3B, C**), consistent with the extensive GA promoted cell expansion occurring at this stage. All four genes also express in floral organs, supporting the development of these tissues, in part, to promote fertility rates (**Figures 3B, C**). The expression of *RrGA3OX1a* and *RrGA3OX2a* in anthesis stage stamens, matches *AtGA3OX1* and *AtGA3OX2* expression in the filaments and anthers (Mitchum et al., 2006; Hu et al., 2008), respectively, and is consistent with the abnormal anther development and poor pollen production of *Arabidopsis ga3ox* mutants and paclobutrazol treated wild radish (**Figures 1E, F** and **2D**). The presence of *RrGA3OX1a* and absence of *RrGA3OX2a* in developing siliques, parallels the same differential expression patterns observed between *AtGA3OX1* and *AtGA3OX2* (**Figures 3B, C**). The presence of *GA3OX* expression in female and immediately adjacent supporting tissues (i.e., receptacle), is consistent with the need for bioactive GA production to promote seed set and silique growth processes (this study and Hu et al., 2008). The apparent exclusive expression of *RrGA3OX1a* in the stem, matches the strongly preferential expression (~100-fold) of *AtGA3OX1* over *AtGA3OX2* (**Figures 3B, C**). This localization is consistent with the requirement of bioactive GA for stem elongation and the resulting dwarfed phenotype of both the *Atga3ox1 Arabidopsis* and paclobutrazol treated wild radish plants (**Figures 1A, B** and **2A, B**). Discrepancies were apparent between the orthologous genes, with expression of neither *RrGA3OX* detected in the leaf nor *RrGA3OX1a* detected in the root, despite both *AtGA3OX1* and *AtGA3OX2* present in these tissues (**Figures 3B, C**). These difference may be due to variation in the exact tissues sampled between studies, for instance; unlike the sampling of whole *Arabidopsis* rosette (Mitchum et al., 2006), we only sampled a hole punch of a fully expanded wild radish leaf blade, which is a developmental stage and region of the leaf where *AtGA3OX1* and *AtGA3OX2* expression begin to dissipate (Mitchum et al., 2006). Alternatively, these discrepancies could indicate the existence of *GA3OX* ohnologs in the wild radish genome.

RrGA3OX1a and RrGA3OX2a were used as queries to mine the genomic sequences and predicted proteomes of wild radish and its close cultivated relative, *Raphanus sativus* (Moghe et al., 2014; Jeong et al., 2016). Six candidate genes from the wild radish genome assembly were identified with sufficient coverage, to be confidently designated as *GA3OX* genes. A similar six *GA3OX* genes were also identified from the independently generated *R. sativus* genome assembly, suggesting that all *Raphanus GA3OX* genes were identified (**Supplementary Figure S6**). *Raphanus* experienced a triplication event since its divergence from *Arabidopsis*, with a ~45% retention of its hexaploid gene complement as it diploidized (Moghe et al., 2014). Assuming the *Arabidopsis*/*Raphanus* common ancestor had four *GA3OX* genes like *Arabidopsis*, then the six identified *Raphanus GA3OX* genes are within expectation [i.e., 4(ancestral genes) x 3(triplication event) x 45%(retention) = 5.4 genes]. Phylogenetic clustering of protein sequences encoded by the two already identified *RrGA3OX* genes and the four from *Arabidopsis*, revealed that the *Raphanus GA3OX* gene family consists of two ohnologs for both *GA3OX1* and *GA3OX2*, along with single copies of *GA3OX3* and *GA3OX4* paralogs (**Figure 3D**). The additional *Raphanus GA3OX* genes were named based on homology with *Arabidopsis GA3OX* sequences and the two originally cloned *RrGA3OX1a* and *RrGA3OX2a* (**Figure 3D**).

### RrGA3OX1a Can Function as a Gibberellin 3-oxidase Converting Inactive GA_9_ Into Bio-Active GA_4_

#### *ga3ox* mutant phenotypic complementation assay

**Figure 4:**
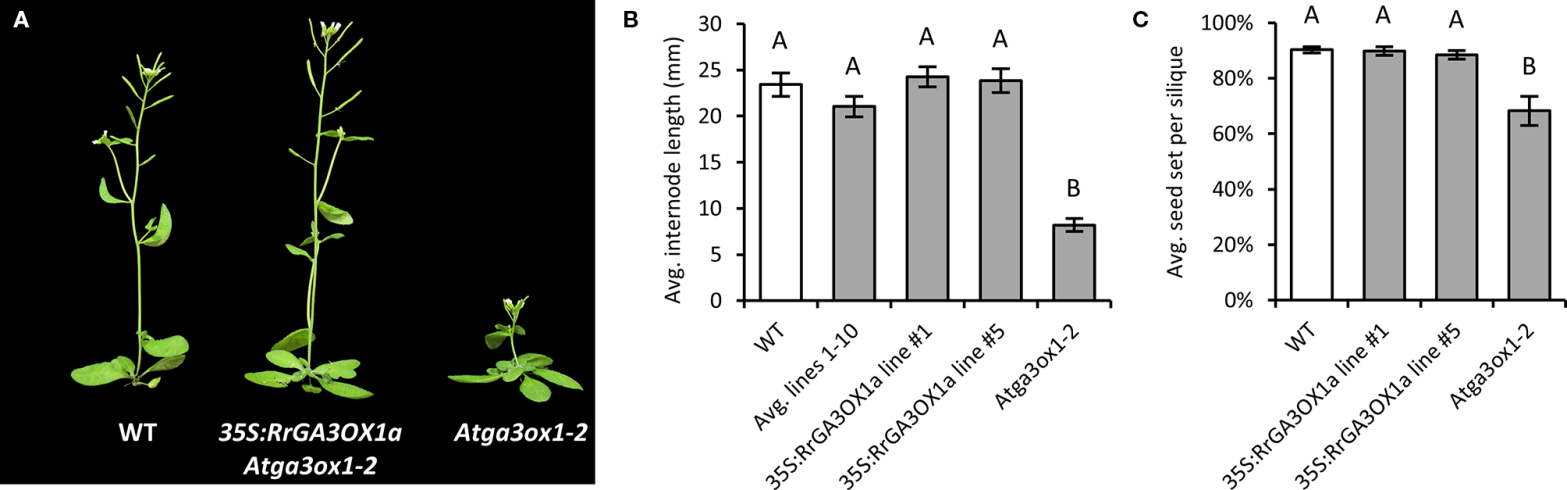
Complementation of the dwarfed stature and reduced fecundity of *Arabidopsis Atga3ox1-2* by over-expressing *RrGA3OX1a* driven by the 35S Cauliflower Mosaic Virus promoter. For confirmation of genotype and transgene expression of the 10 independent *35S:RrGA3OX1a Atga3ox1-2* complemented lines, see **Supplementary Figure S7**. **(A)** A qualitative example showing the improved growth stature of a *35S:RrGA3OX1a Atga3ox1-2* transgenic plant (line #1) over its *Atga3ox1-2* progenitor. **(B)** An assessment of average internode length quantifying the improved primary bolt elongation of the *35S:RrGA3OX1a Atga3ox1-2* transgenic lines. The data is presented as an average of all 10 independent transgenic lines (i.e., one random T_2_ transgenic plant from each line) as well as measurements from two higher expressing complemented lines (lines #1 and #5). N = 10 (WT); 10 (Avg. lines 1–10); 5 (line #1); 5 (line #5); 10 (*Atga3ox1-2*). There was no significant difference between the number of nodes to first flower between wild type (WT) and the complemented lines; 3.5 ± 0.1 (WT), 3.5 ± 0.2 (Avg. lines 1–10); 3.4 ± 0.2 (line #1); 3.2 ± 0.2 (line #5). *At3ox1-2* produced fewer node; 2.6 ± 0.2 (Student’s *t*-test p < 0.05). **(C)** *35S:RrGA3OX1a* rescues the reduced fecundity of *Atga3ox1-2*. Seed set per silique = seed/(seed + unfertilized ovules). N = 10 siliques for each genotype. There was no significant difference in the number of ovules per pistil produced by the different genotypes; 45.90 ± 1.49 (WT); 45.80 ± 0.93 (line #1); 44.20 ± 1.48 (line #5); 48.20 ± 1.44 (*Atga3ox1-2*) (Student’s *t*-test p > 0.05). Letters above bars in **(B**, **C)** represent a pairwise statistical comparison between categories. Categories marked with a given letter are statistically different from categories marked with another letter (Student’s *t*-test p < 0.05). Error bars are S.E.M.

RrGA3OX1a was selected as the representative wild radish GA 3-oxidase for functional testing to confirm GA 3β-hydroxylase activity (i.e., conversion of inactive precursor GA_9_ into the biologically active GA_4_). GA 3-oxidase functionality was first tested by transforming *Atga3ox1-2* mutant plants with a constitutively over-expressing version of *RrGA3OX1a*. Ten independent T_1_ transgenic lines were generated and confirmed in the T_2_ generation to be *Atga3ox1-*2 homozygous mutants expressing *RrGA3OX1a* (**Supplementary Figures 7A, B**). Examination of the T_2_ lines revealed that transgenic expression of *RrGA3OX1a* was capable of rescuing the characteristic dwarfed phenotype (**Figures 4A, B**; **Supplementary Figure 7C**), as well as the reduced fecundity (**Figure 4C**; **Supplementary Figure 7D**), of the *Atga3ox1-2* mutant. This suggests that RrGA3OX1a likely has the capacity to catalyze the conversion of GA_9_ to GA_4_.

#### Novel Yeast Assay for Testing Gibberellin 3-oxidase Activity

**Figure 5:**
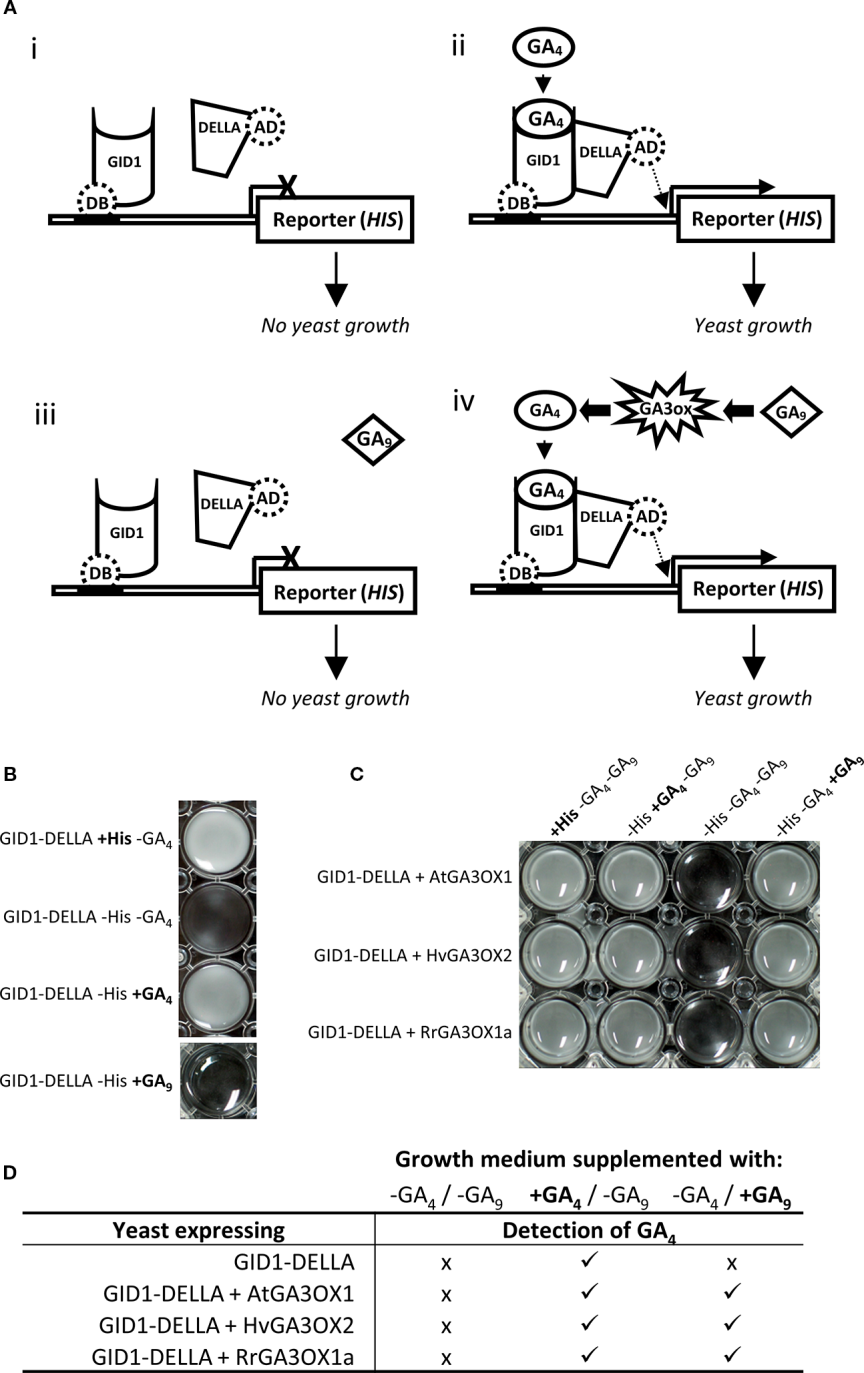
Functional confirmation of the ability for RrGA3OX1a to convert inactive GA_9_ into active GA_4_ using a novel modification to the GID1-DELLA yeast two-hybrid system. **(A)** A graphical representation of the yeast two-hybrid system. (i-ii) Basic GID1-DELLA system. The GID1 protein is translationally fused to a DNA binding (DB) domain enabling it to bind to the promoter of the histidine (*HIS3*) reporter gene. The DELLA protein is translationally fused to a transcriptional activation domain (AD), that when brought into close proximity of the *HIS3* gene by interaction with GID1, promotes *HIS3* transcription. Interaction of GID1 and the DELLA proteins only occurs in the presence of GA_4_ which results in transcriptional activation of the *HIS3* reporter gene, allowing the histidine auxotrophic yeast to grow in the absence of exogenously supplied histidine (iii-iv) Incorporating a *GA3OX* into the GID1-DELLA yeast system allows pre-cursor GA_9_ to be fed to the yeast and the ability of the GA3OX to convert GA_9_ to GA_4_ assayed through interaction of GID1-DELLA and subsequent activation of the *HIS3* reporter gene and growth of the yeast culture. **(B)** Aliquots of yeast cultures showing that the basic GID1-DELLA yeast growth is reliant on the supply of exogenous histidine or expression of the *HIS3* reporter which is dependent on GA_4_ induced GID1-DELLA interaction. **(C)** Aliquots of yeast cultures showing that co-expression of *GA3OX* in the yeast allows the system to function through GA_9_. *RrGA3OX1a* expressing yeast shows similar growth responses to both GA_9_ and GA_4_ feeding as seen with *AtGA3OX1* and *HvGA3OX2;* indicating that *RrGA3OX1a* encodes a functional GA 3-oxidase. **(D)** Gas chromatography–mass spectrometry (GC-MS) detection of GA_4_ in yeast cultures, confirming the conversion of GA_9_ to GA_4_ in the *GA3OX* expressing yeast. (−) medium not supplemented with GA_4_ or GA_9_. (+) medium supplemented with GA_4_ or GA_9_. (x) GA_4_ not detected. () GA_4_ detected.

In plants, the interaction between the GA receptor (GID1) and DELLA proteins occurs due to a conformational change in GID1 induced by binding a “biologically active” GA, such as GA_4_ (Ueguchi-Tanaka et al., 2005). This GA_4_-GID1-DELLA interaction can be recreated heterologously in yeast using the yeast two-hybrid (Y2H) system and assayed through either inactivation (GA_4_ absent) or activation (GA_4_ present) of reporter genes (Ueguchi-Tanaka et al., 2007). We modified this system to provide a novel means of directly testing the GA3OX capability of RrGA3OX1a (**Figure 5A**). The rice GA receptor (GID1) and DELLA (SLENDER1; SLR1) proteins were expressed as a Y2H DNA binding bait and transcriptional activating prey, respectively, in a histidine auxotrophic yeast stain carrying a histidine (HIS3) reporter gene driven by a promoter recognized by the DNA binding domain fused to GID1. In this set up, the histidine auxotrophic yeast strain can only grow in medium lacking the amino acid histidine if the culture is supplemented with GA_4_ to initiate GID1-DELLA interaction and subsequent transcriptional activation of the *HIS3* reporter gene (**Figures 5Ai-ii**).

The GID1+DELLA containing yeast was confirmed to require exogenous supplementation of histidine in order to grow (**Figure 5B**). In the absence of exogenous histidine, supplementing with GA_4_ could complement the HIS deficiency by allowing GID1-DELLA interaction and activation of the *HIS3* reporter gene (**Figure 5B**). There was no detectable GID1-DELLA interaction when the yeast cultures were supplemented with bio-inactive GA_9_ (**Figures 5Aiii, B**). However, when we introduced a third transgene into the yeast that encodes for a GA3OX protein from either *Arabidopsis* or barley, which have confirmed GA 3β-hydroxylase activity (Williams et al., 1998; Spielmeyer et al., 2004; Suzuki et al., 2005; Pearce et al., 2015), the GA_9_ supplemented yeast cultures grew. This implies the yeast were synthesizing GA_4_ from the GA_9_ which in turn enabled the GID1-DELLA interaction, leading to activation of the *HIS3* reporter gene and allowing the HIS deficient yeast to grow (**Figures 5Aiv, C**). Gas chromatography–mass spectrometry (GC-MS) analysis of the cultured supernatants of yeast supplement with GA_9_ detected the presence of GA_4_ in only the *GA3OX* expressing yeast lines, confirming the conclusions derived from the GA3OX GID1-DELLA Y2H system (**Figure 5D**). Similar results were obtained with RrGA3OX1a, demonstrating its capability to function as a GA3OX (**Figures 5C, D**).

### Wild Radish and Wheat GA3OX Proteins Differ in Predicted Substrate Specificity Defining Residues

To possibly develop discriminating GA3OX inhibitory compounds, significant differences must exist in the GA_n_ recognition region of GA3OX enzymes between the dicot wild radish and monocot wheat. However, evaluating if such difference occur is complicated by the uncertainty of which residues determine GA_n_ variant recognition for any of the GAOX enzymes. To narrow down to the level of candidate residues to compare between monocot and dicot GA3OX proteins, we first needed to localize protein region(s) likely responsible for differential GA_n_ recognition. We hypothesized that this could be achieved by comparing the GA20OX, GA3OX, and GA2OX classes that form similar gross protein structures but would have key differences allowing recognition of their distinct preferred GA_n_ variants.

#### GAOX Proteins Appear Functionally Conserved Yet Suitably Diverse Between Monocot and Dicots

**Figure 6:**
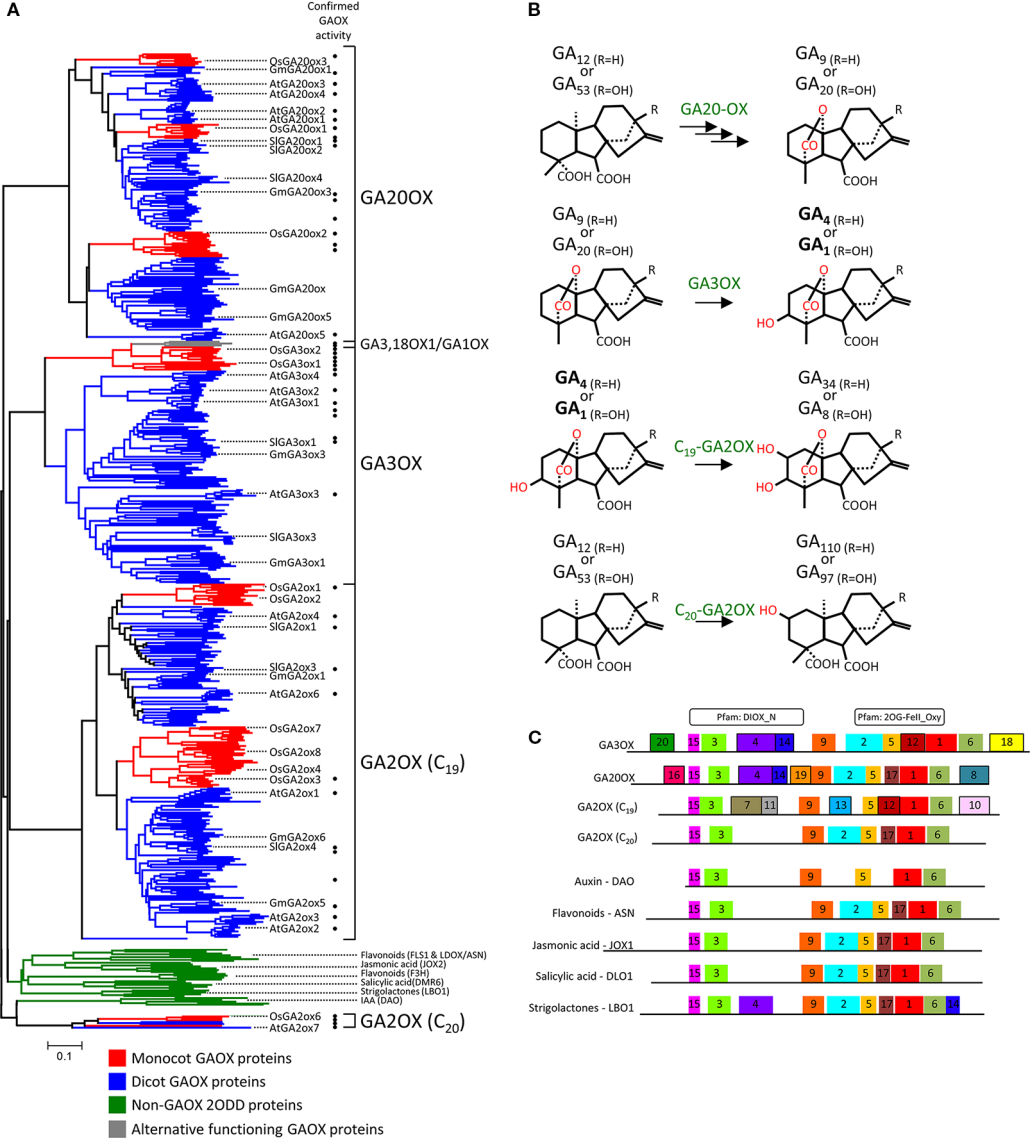
Phylogenetic characterization of GA-OXIDASE proteins **(A)** Phylogenetic relationship of 482 GAOX and 34 related non-GAOX 2ODD protein sequences from across 37 angiosperm species. The non-GA3OX 2ODDs represent 2ODD enzymes that function in the biosynthesis or deactivation of flavonoids, jasmonic acid, salicylic acid, strigolactones, or auxin. The phylogeny is separated into five major clades, four of which represent the GA20OX, GA3OX, C_19_-GA2OX, and C_20_-GA2OX classes of the GA-OXIDASE (GAOX) family. The GAOX sequences clearly segregate from the non-GAOX 2ODD representative group (green clade), indicating they are a distinct group of 2ODD proteins. GAOX functionality was confirmed by cross-matching against reported biochemical analysis, which confirmed the expected specific GAOX activity (i.e., GA20OX, GA3OX, or GA2OX GA modifications) for numerous members from across the phylogeny (solid circles to the right of the branches; **Supplementary Table S6**). Representative GAOX members from major divisions of angiosperms are marked to assist navigating the compressed phylogeny (monocot, rice, *Oryza sativa*, Os; dicot, Malvidae, *Arabidopsis*, *Arabidopsis thaliana*, At; dicot, Fabidae, soybean, *Glycine max*, Gm; dicot, asterid, tomato, *Solanum lycopersicum*, Sl). Monocot (red clades) and dicot species (blue clades) are present in each of the GAOX classes but cluster separately from each other, suggesting significant independent diversification between monocot and dicot GAOX proteins. Substantial intra-order diversification is also apparent and can be examined with the expanded format of this phylogeny (**Supplementary Figure S8**). For details of tree generation, see *Experimental Procedures*. The plant species from which the 482 GAOX proteins were obtained are summarized in **Supplementary Table S1**. The protein sequences used in the alignment and generation of the phylogenetic tree are found in **Supplementary Tables S2**, **S3**, **S4**. Scale bar represents frequency of amino acid substitutions per site. **(B)** Gibberellin modifications catalyzed by the GA20OX, GA3OX, and GA2OX (C_19_ and C_20_) class of GAOX enzymes. Depicted are the inactive precursor variants that are converted by GA20OX and then GA3OX to synthesize bioactive GA_1_ or GA_4_ (bold), which can then be converted to inactivate catabolites by GA2OX. C_20_-GA2OX enzymes inhibit bioactive GA production by metabolizing C_20_-GA precursors. **(C)** Distribution of the conserved protein motifs identified using MEME. Represented are the consensus motif patterns for the different 2ODD protein groups. For MEME motif pattern of each individual gene see **Supplementary Figure S13**. Above; location of the two major Pfam motifs that identify 2ODD proteins.

We began by exploring the diversity of plant GAOX enzymes to gauge the suitability of our approach. GAOXs are members of a larger enzyme family known as 2-oxoglutarate-dependent dioxygenase (2ODD), that are present across all taxonomic kingdoms (Islam et al., 2018). 2ODDs are extensively diverse in plants and catalyze a remarkably diverse range of oxidative reactions (Farrow and Facchini, 2014; Kawai et al., 2014). Sequences from 482 2ODD proteins annotated as GAOX enzymes were retrieved from across 37 angiosperm species (**Supplementary Tables S1**–**S4**). A further 34 sequences of the most closely related but functionally distinct 2ODD enzymes were also included for comparison. Orthologs of these non-GAOX 2ODD representative group were retrieved from two diverse monocots (rice and *Brachypodium*) and dicot (*Arabidopsis* and tomato) species, and have confirmed functions in the biosynthesis or deactivation of flavonoids, jasmonic acid, salicylic acid, strigolactones, or auxin (Wisman et al., 1998; Wilmouth et al., 2002; Owens et al., 2008; Brewer et al., 2016; Zhang et al., 2016; Caarls et al., 2017; Zhang et al., 2017) (**Supplementary Tables S5** and **S6**). All 516 sequences were aligned and phylogenetically compared (**Figure 6A** and **Supplementary Figure S8**). The phylogeny separated into several distinct clades, with the putative GAOX proteins clearly segregated from the non-GAOX 2ODD representative group (**Figure 6A**). We validated our distinct group of 2ODD proteins as GAOX proteins by cross-matching against reported *in vitro* assays. This confirmed the expected GA20OX, GA3OX, or GA2OX (C_19_ and C_20_) activity of numerous members from across the phylogeny (**Figure 6A**; **Supplementary Table S6**).

Being of the same enzyme family, there is a degree of homology between all the GAOX proteins that allows them to form similar tertiary structures in order to recognize and execute modifications on GA molecules. However, when the GAOXs are compared phylogenetically, the protein sequences distinctly group into three major large clades comprising the GA20OX, GA3OX, and C_19_-GA2OX enzymes classes (**Figure 6A** and **Supplementary Figure S8**). A forth, smaller clade is populated by C_20_-GA2OXs (**Figure 6A**). These GA2OXs inhibit bioactive GA production by metabolizing C_20_-GA precursors and appear evolutionarily related to IAA catabolizing 2ODD proteins (**Figures 6A, B**). The discrete phylogenetic segregation reflects the functional differences between the four classes of GA biosynthetic enzymes, which includes GA variant recognition and structural modifications to specific carbon atoms of the GA ring structures (**Figure 6B**). Monocot and dicot sequences are present in each of the three major GAOX clades, suggesting that protein sequences related with function are sufficiently conserved to produce clustering of functional classes rather than being overly obscured by evolutionary lineages. This is important for our investigative approach as it indicates that sequence comparisons between the major clades could indeed highlight regions and even potentially specific candidate residues distinguishing between the preferred GA_n_ variants of the different GAOX classes.

MEME analysis of the 516 sequences identified a series of motifs conserved in sequence and position among the GAOX and non-GAOX 2ODD proteins (**Figure 6C**). Many of the motifs are common across all groups and overlap general consensus Pfam motifs identifying 2ODDs, implicating them as integral functional units fundamental to general 2ODD activity. Several motifs are predominantly (motifs 4 and 14) or exclusively (motifs 7, 8, 10, 11, 12, 13, 16, 18, 19, 20) present in GAOX proteins, suggesting these likely define GAOX-specific activity. The different assortment of these variable motifs among the GA20OX, GA3OX, and GA2OX proteins may then specify the different capabilities of the GAOX classes (i.e., GA recognition and modification). The motif set of 4, 14, 18, and 20 is most intriguing with regards to our class of interest, the GA3OX proteins (**Figure 6C**).

The phylogeny further showed that within a GAOX major clade, the monocot orthologs cluster separately from their dicot counterparts (**Figure 6A**). Distinct sub-clades of monocot and dicot sequences occur in the GA20OX and GA2OX clades, indicating the emergence of discrete isoforms prior to the divergence of monocots and dicots (**Figure 6A**). Since the monocot-dicot split, further substantial duplication and diversification of GAOX proteins is apparent between orders of dicots and between major clades of monocots as evident by species clustering patterns (**Supplementary Figure S8**). This internal division reveals significant independent diversification of GAOX proteins between monocots and dicots. This is equally true for the GA3OX proteins, which bring with it the possibility of even minor variation to substrate specificity between monocot wheat and dicot wild radish that could result in differential affinity for competitive inhibitory molecules.

#### Modeling Wild Radish and Wheat GA3OX Proteins to Predict Functionally Important Residues

**Figure 7:**
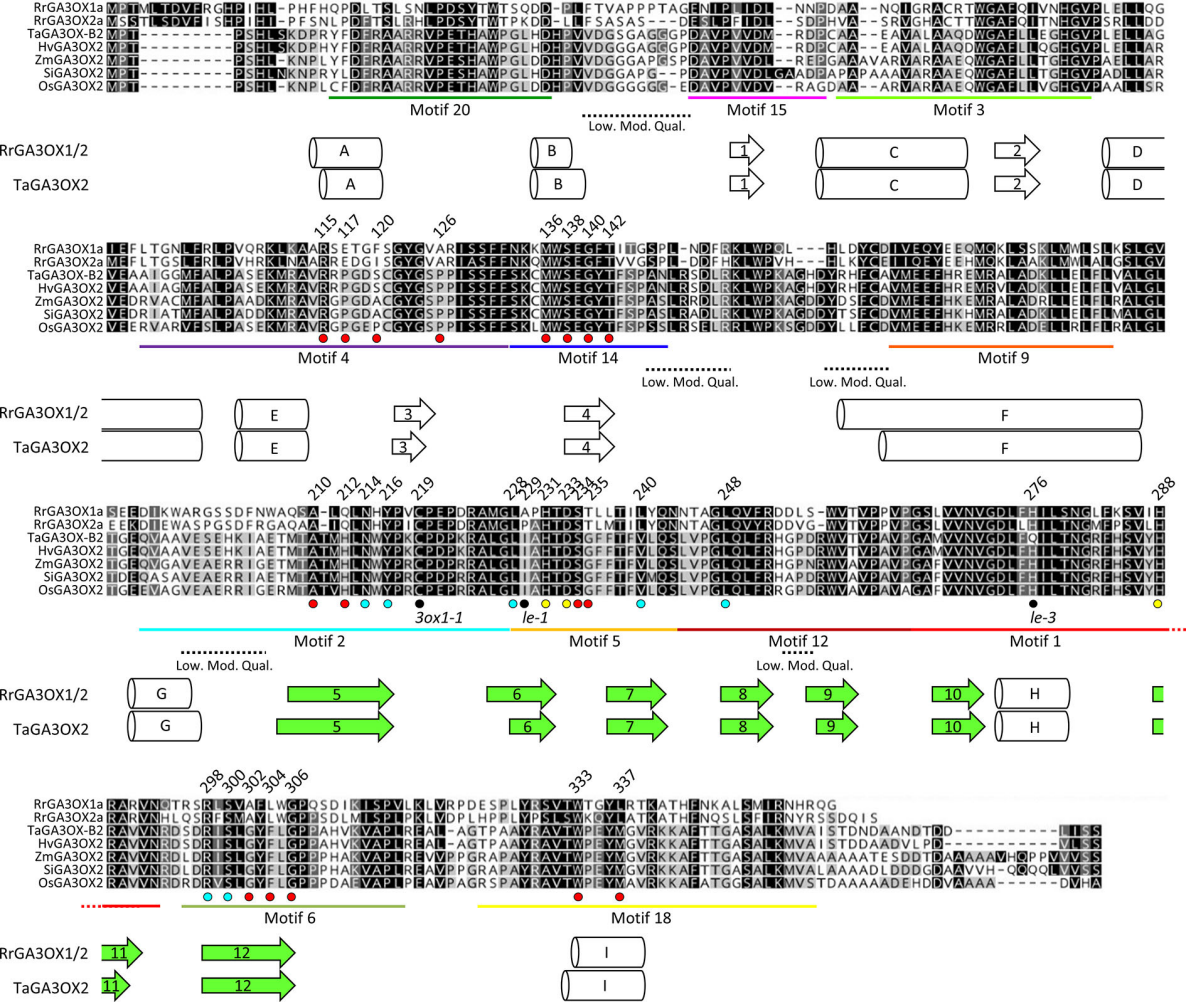
Sequence alignment comparing RrGA3OX1a and RrGA3OX2a against TaGA3OX2 and its orthologs from several other monocot species. MUSCLE generated protein sequence alignment. Shading denote amino acids with similar properties (based on ≥1 Blosum62 matrix score) and their prevalence across the seven sequences (black 100%; dark gray 80–100%; light grey 60–80%; white < 60%). Predicted secondary structures underlie the involved residues (arrows = β-strands; cylinders = α-helices). β-strand secondary structures 5–12 (shaded green) form the “jelly-roll” motif reaction cavity in which docks the co-factor FE(II) ion, 2-oxoglutarate co-substrate (2OG), and the bio-inactive GA precursor molecule. MEME identified sequence motifs from **Figure 6C**, are underlined, labeled, and color matched in accordance with **Figure 6C**. Substrate contact residues identified in **Figures 8E, F** are indicated by dots below the sequence alignment. Cyan dots = 2-oxoglutarate binding residues. Yellow dots = Fe ion binding residues. Red dots = 12 residues predicted to be capable of making contact with the docked GA substrate. Additional residues of interest; black dots = residues altered in *Arabidopsis* (*3ox1-1*) and pea (*le* and *le-3*) *ga3ox* mutants. Dashed lines underline regions of lower modeling quality (Low. Mod. Qual.); i.e., higher root-mean-square deviation (RMSD) in atomic positions between the RrGA3OX1a and TaGA3OX-B2 protein tertiary structures with the modeling template.

To evaluate if the diversity between monocot and dicot GA3OX proteins occur at positions potentially involved in GA interaction that may be exploited for species-specific GA3OX inhibition, we compared the structures of RrGA3OX1a, RrGA3OX2a, and wheat GA3OX proteins. Wheat has two GA3OX proteins (TaGA3OX2 and TaGA3OX3; Ta—*Triticum aestivum*), each encoded by three homologs (*TaGA3OX-A2*, -*B2*, -*D2*, and *-A3*, -*B3*, -*D3*). Both are confirmed to mainly function as 3β-hydroxylases (Appleford et al., 2006). However, *TaGA3OX3* is expressed exclusively in the grain, with only *TaGA3OX2* being broadly expressed in vegetative and reproductive tissues (Pearce et al., 2015). Therefore, we compared RrGA3OX1a and RrGA3OX2a against TaGA3OX2 and its orthologs from several other monocot species. Wild radish and monocot GA3OX proteins share between 52 and 56% similarity in their protein sequences and have a strong conservation in their secondary structures (**Figure 7**). Both the GA3OX proteins from wild radish and wheat, are predicted to comprise of 8 α-helical and 12 β-strands motifs at equivalent locations within the protein sequence (**Figure 7**).

**Figure 8:**
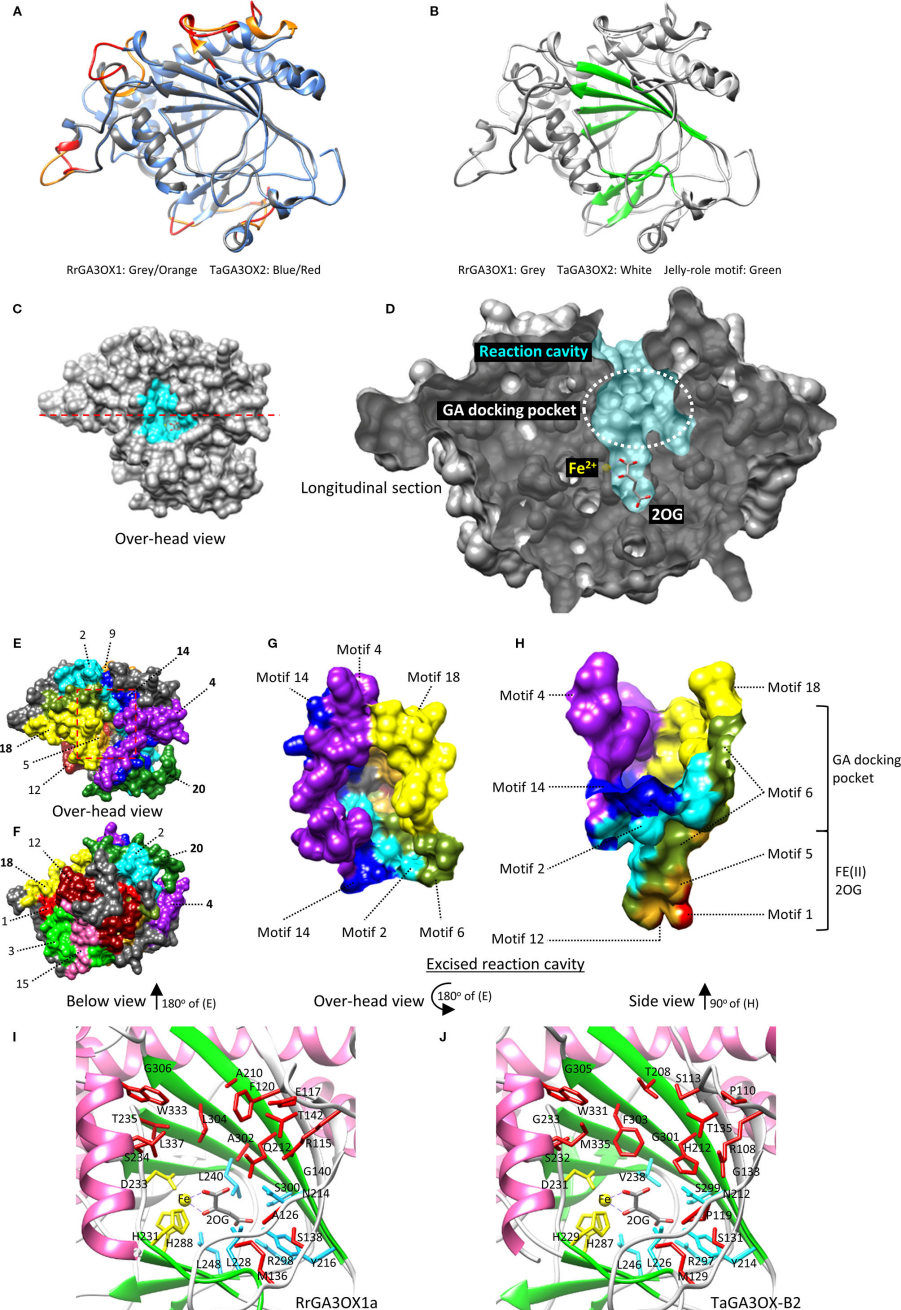
Modeling wild radish and wheat GA3OX proteins to identify functionally important regions and potential substrate contact residues. **(A)** Tertiary protein structure of RrGA3OX1 superimposed over TaGA3OX1. The two proteins have very similar modeled structures with regions of high similarity depicted in grey/blue. Regions with greater structural differences between the two proteins are colored red/orange (“Low. Mod. Qual.” in **Figure 7**). **(B)** Superimposed tertiary structures of RrGA3OX1 and TaGA3OX1 proteins highlighting the well modeled jelly-roll motif which constitutes the reaction cavity. **(C, D)** Surface mapping of the RrGA3OX1a tertiary protein structure reveals the reaction cavity; similar for all RrGA3OX and TaGA3OX proteins. **(C)** Over-head vantage providing a view into the reaction cavity (highlighted cyan). **(D)** Longitudinal section along the red dashed line in **(C)**, revealing the profile of the reaction cavity. In the depths of the reaction cavity residue the FE(II) ion and 2OG co-substrate. Superior to these molecules is the GA docking pocket. **(E, H)** Mapping MEME motifs onto the RrGA3OX tertiary structure. **(E)** Similar overhead vantage view as in **(C)**, but with the residues color coded according to their residing motif. Color coding and number labels match those of the MEME output in **Figure 6C**. **(F)** Below view (180° rotation of (E) pivoting on the x-axis) providing another perspective and revealing the other motifs. **(G, H)** A dissection of the tertiary structure showing the excised reaction cavity and the motif sequences that comprise it. **(G)** Over-head view looking down into the reaction cavity. **(H)** Side view of the intact reaction cavity showing the contribution of the different motifs to both the GA docking pocket and the deeper FE(II) and 2OG docking pocket. The GA docking pocket is made up of motifs 2, 4, 6, 14, and 18. The depths of the cavity where the FE(II) and 2OG reside are made up of motifs 1, 2, 5, 6, and 12. **(I, J)** Close-up of the reaction cavity of both RrGA3OX1a and TaGA3OX1-2, showing the amino acids lining the cavity with reactive sidechains orientated inwards toward the reactive center. The position of the FE(II) ion is coordinated by side chains of yellow colored residue, while the position of the 2OG co-substrate is co-ordinated by the cyan colored residues. The 12 putative GA contact residues, surrounding the GA docking pocket (black dashed circle), are colored red.

2ODD enzymes in general, can have as little as 20% sequence homology and still produce very similar tertiary structures (Wilmouth et al., 2002; Kluza et al., 2018). Homology modeling has been successfully used to accurately predict essential residues involved in differential substrate recognition by 2ODD enzymes. Examples include, flavanone 3b-hydroxylase (FHT) *vs.* flavone synthase I (FNSI) and feruloyl-CoA 6′-hydroxylase (F6′H1) *vs.* p-coumaroyl CoA 2′-hydroxylase (C2′H), which are involved in flavonoid and coumarin biosynthesis, respectively (Gebhardt et al., 2007; Sun et al., 2015). Both of these studies involved closely related 2ODDs that differentiate between slightly different precursor substrates; a scenario comparable to that of the GAOX classes. The target-template sequence similarity in these studies was ~30%, which is comparable to sequence similarity between the RrGA3OX and TaGA3OX proteins and the best available 2ODD crystal structure template options in the Protein Data Bank (PDB: https://www.rcsb.org/). Given this precedent, we used SWISS-MODEL to generate tertiary structures of the RrGA3OX and TaGA3OX enzymes using the resolved crystal structures of 2ODD enzymes *Arabidopsis* ANTHOCYANIDIN SYNTHASE (AtANS; PDBID: 1GP4, 1GP5, and 1GP6) (Wilmouth et al., 2002) and *Papaver somniferum* (Poppy) THEBAINE 6-O-DEMETHYLASE (T6ODM; PDBID: 5o7y, 5o9w) (Kluza et al., 2018) as templates (**Figures 8A, B**; **Supplementary Figure S9**). The RrGA3OX and TaGA3OX proteins share between 26 and 32% sequence identify with the templates, and were modeled with good accuracy as determined using root-mean-square deviation (RMSD), global quality estimation (GMQE), and qualitative model energy analysis scores (QMEAN) (Waterhouse et al., 2018). Only small regions found on surface loops showed lower modeling quality (**Figures 7** and **8A**; **Supplementary Figure S9**), but even these appear orientated well with conserved hydrophobic faces directed inwards toward the protein center and hydrophilic faces exposed to the surrounding solvent. The core “jelly-roll” motif, which is the characteristic reaction center of all 2-ODD enzymes, including GAOX proteins (Aik et al., 2012), was modeled with high accuracy (**Figures 8A, B**; **Supplementary Figure S9**). This core region is predominantly comprised of β-strand motifs 5–12 and forms the foundation of the reaction cavity in which docks the co-factor FE(II) ion, 2-oxoglutarate co-substrate (2OG), and the bio-inactive GA precursor molecule. Key functional residues, including those determining specificity and affinity for different GA_n_ variants, will be contained within this cavity.

Surface mapping of the protein structure revealed more prominently the reaction cavity of the GA3OX proteins (**Figures 8C, D**). The FE(II) ion and 2OG co-substrate reside in the depths of the cavity (**Figures 8C, D**), which is formed by parts of the MEME protein sequence motifs 1, 2, 5, 6, and 12 (i.e., the common 2ODD motifs) (**Figures 8E, F, G, H**). The FE(II) and 2OG positions are coordinated by a series of residues strictly conserved between our homology models and the templates, and are highly conserved among GAOXs and 2ODD enzymes in general (Wilmouth et al., 2002; Sim et al., 2003; Seo et al., 2004; Chua et al., 2008; Kluza et al., 2018) (depicted in yellow for FE(II) and cyan for the 2OG co-substrate in **Figures 7** and **8I, J**). The location of the GA molecule within the reaction cavity was defined by homologous residency of dihydroquercetin, ferulic acid, and the baine within ASN, F6′H1, and T6ODM, respectively (**Figure 8D**) (Wilmouth et al., 2002; Sun et al., 2015; Kluza et al., 2018). The protein regions directly forming the GA binding pocket, and therefore specifying GA variant recognition and modifications, include motifs 2 and 6, but predominantly motifs 4, 14, and 18; which are three of the four motifs uniquely characteristic of GA3OX proteins (**Figures 8G, H**). Even the final member of the GA3OX-specific motif set, the distal N-terminal motif 20, appears to help enclose the GA binding pocket by wrapping around the back face of the cavity and intertwining between motifs 2 and 14 (**Figures 8E, F**). Their involvement in forming the GA binding pocket, strongly implicates that these GA3OX-specific motifs, and their unique counterparts at equivalent locations in GA20OX and GA2OX proteins, especially contribute to defining the different GA specificity and activity of the GAOX class.

To get to single amino acid level, we identified all residues lining the GA reaction pocket, as among them must be the specificity defining residues determining GA_n_ variant recognition. Of these, 23 residues were flagged as possibilities for substrate interactions based on similar positional properties of contact residues from other 2ODD proteins (Roach et al., 1997; Lee et al., 2001; Wilmouth et al., 2002; Zhang et al., 2002; Gebhardt et al., 2007; Sun et al., 2015; Chowdhury et al., 2016; Kluza et al., 2018). These 23 residues fit a criteria of being within 5Å of a substrate in the docking pocket, with reactive sidechains orientated inwards toward the reactive center and the docked GA (**Figures 7** and **8I, J**). Six of these residues are among those involved in orientating the FE(II) and 2OG co-substrate (**Figures 8I, J**), and occur in motifs 1, 2, 5, 6, and 12 (**Figure 7**). This leaves 17 residues within a plausible contact distance of a residing GA molecule (**Figures 8I, J**). This group of 17 possible GA interaction residues are the stronger candidates, from among those lining the GA pocket, for defining GA_n_ variant specificity and orientation toward the FE(II) and 2OG co-substrates. Ten of these 17 residues, occur within the GA3OX-characteristic motif set (**Figure 7**).

#### Comparing Sequence Differences Between Monocot and Dicot GA3OX Proteins

Having isolated the most relevant protein regions and residues pertaining to possible GA_n_ recognition, we could now evaluate these between GAOX classes and more specifically monocot and dicot GA3OXs. We compared multiple sequence alignments of GAOX proteins, examining conservation between and within classes and sub-classes, for indicators of positions and residues of functional importance.

Residues at positions interacting with the FE(II) ion and the 2OG co-substrate, are essentially invariable between the classes, consistent with their involvement in the core catalytic function of the GAOX proteins (**Supplementary Figure S10**). The GA3OX motifs that form the GA docking pocket share a number of highly conserved residues with their positional counterparts in GA2OX and GA20OX (**Supplementary Figure S10**). These are likely key structural residues essential for organizing the GAOX reaction cavity and generic GA_n_ interactions. The positionally homologous motifs also exhibited highly conserved polymorphisms, often involving amino acids with different biochemical properties, which differentiate the three GAOX classes. These differentially conserved positions/residues are good candidates for defining GA_n_ specificity (**Supplementary Figure S10**).

**Figure 9:**
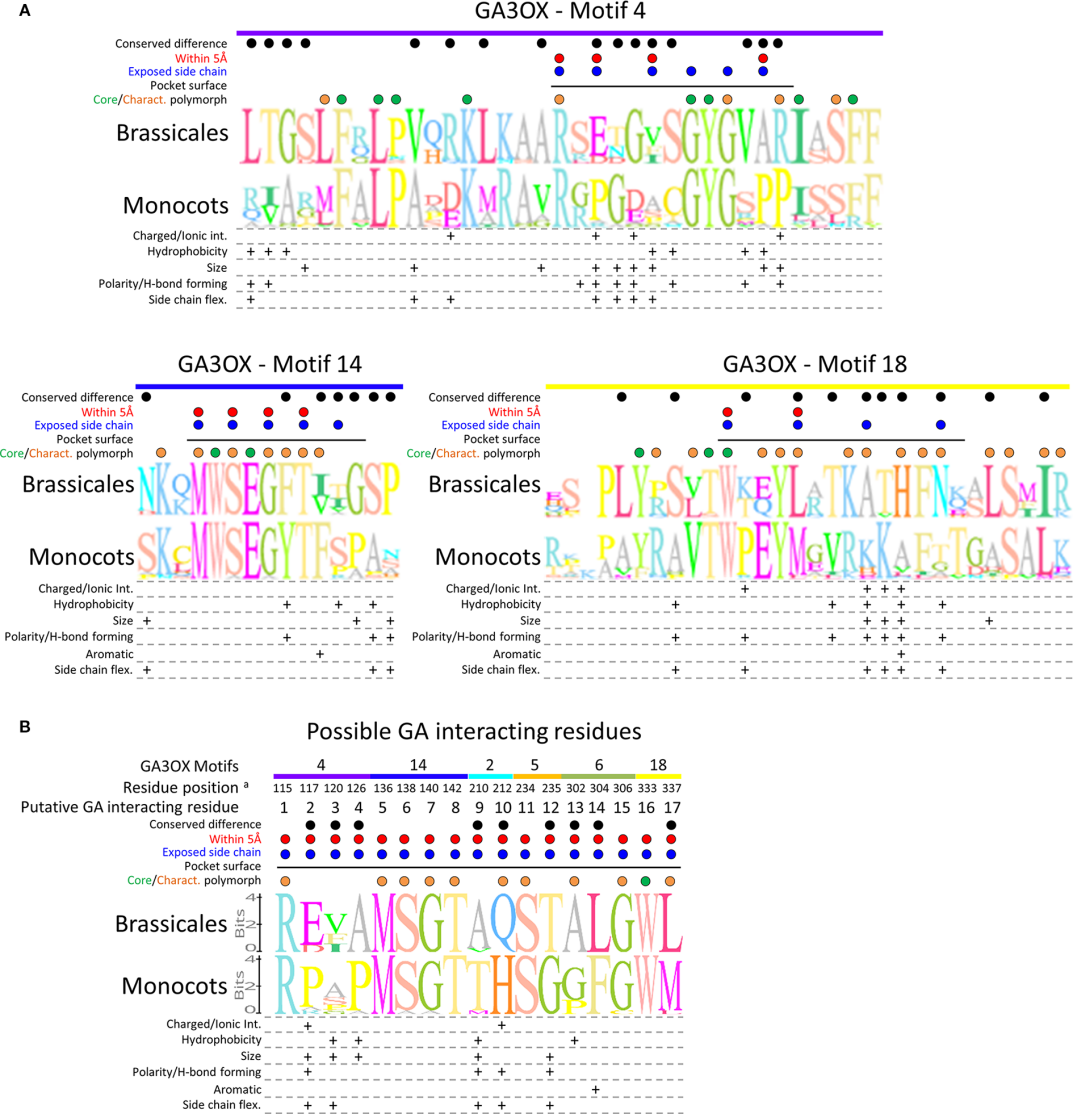
Comparing protein sequences of the GA docking pocket between monocots and Brassicales GA3OX proteins. **(A)** Sequence logo alignments comparing the GA3OX-specific motifs forming the GA docking pocket, with differences in biochemical properties of the conserved substituted amino acids below. **(B)** Sequence logo alignments comparing the putative GA contact residues within the GA3OX GA docking pocket, with differences in biochemical properties of the conserved substituted amino acids below. Sequence logos: the overall height of the residue logo stack indicates the sequence conservation at that position, whereas the height of the given residue reflects the relative frequency of the corresponding amino acid. The motifs from which each of the interacting residues originate are indicated along the top of the alignments; color coding and motif labels are concordant with **Figure 6C**. Dots above the sequences: green—core residues conserved across all GAOX classes, orange—conserved polymorphisms characteristic (Charact.) of GA3OX proteins, blue—pocket lining residues with reactive side chains orientated inwards toward the substrate, red—same as blue marked residues, except within 5Å of docked GA molecule, black—conserved polymorphic residues between monocot and Brassicales. Black solid horizontal line: Residues of the motif that line the interior surface of the GA docking pocket. Amino acid property groups used to characterize the biochemical properties of the conserved substituted amino acids: charged resides (capable of forming ionic interactions): positive [R, H, K], negative [D, E], uncharged [A, N, C, Q, G, I, L, M, F, P, S, T, W, Y, V]. Hydrophobicity: hydrophobic [I, L, V, C, A, M, F], neutral/hydrophilic [G, Y, W, H, K, T, R, E, Q, D, N, S, P]. Size: small [A, G, C, S], medium [V, T, N, P, D], large [Q, E, H, K, R, F, Y, W, M, I, L]. Polarity (capable of forming H-bonds): charged polar [H, R, K, E, D], uncharged polar [Q, T, S, N, Y, W], non-polar [A, G, V, L, I, P, F, M]. Side chain flexibility: high [K, E, M, Q, R], moderate [D, F, H, I, L, N, W, Y], low [C, S, T, V], limited [A], restricted [P]. Sequences: monocot GA3OXs; TaeGA3OX-B2 (wheat), HvuGA3OX2 (barley), Sit5G125501 (Setaria), PvirJ243921 (Panicum), Sbi003G0459001 (Sorghum), Msi05G0336001 (Miscanthus), ZmaNP_001266453 (Maize), OsaGA3OX2 (rice), Bdi2G04840 (*Brachypodium*), OsaGA3OX1 (rice), Sit025127 (Setaria), ZmaNP_001146525 (Maize), and Sbi005400 (Sorghum). Brassicales GA3OXs; RrGA3OX1a (wild radish), Bra026757 (Brassica rapa), RrGA3OX1b (wild radish), Bra026122 (Brassica rapa), Tha008008 (Thellungiella halophile), Cru0009532 (Capsella rubella), Aly471756 (Arabidopsis lyrata), AthGA3OX1 (*Arabidopsis thaliana*), AthGA3OX2 (*A. thaliana*), Aly909680 (*Arabidopsis lyrata*), RrGA3OX2a (wild radish). For sequences, see **Supplementary Tables S2**–**S4**. For their phylogenetic relationships, see **Supplementary Figure 8**. ^a^Residue position in relation to the RrGA3OX1a sequence in **Figure 7**.

As expected, the core structural residues and the majority of the GA3OX characteristic polymorphisms, were found to be identical, or at least conservative biochemical substitutions, between monocot and dicot GA3OX proteins. This holds true for our specific comparison of interest between GA3OX proteins from monocots (e.g., wheat) and dicot Brassicales (e.g., wild radish) (**Figure 9A**). Despite the higher conservation, conserved polymorphisms were still present between the monocots and Brassicales GA3OX proteins. In some instances, these occurred at GA3OX characteristic positions and/or residues forming the surface of the GA binding pocket, and often involve substitutions of amino acids with different biochemical properties (**Figure 9A**). Eight such polymorphisms were found to specifically occur among the 17 possible GA interacting residues (**Figure 9B**). Collectively the results indicate that the GA docking pockets of wheat and wild radish, likely have sufficiently different interaction possibilities to reasonably expect differential affinity for competitive inhibitory molecules.

### *DELLA Overgrowth* Mutants in Barley Show Resistance to Paclobutrazol

**Figure 10:**
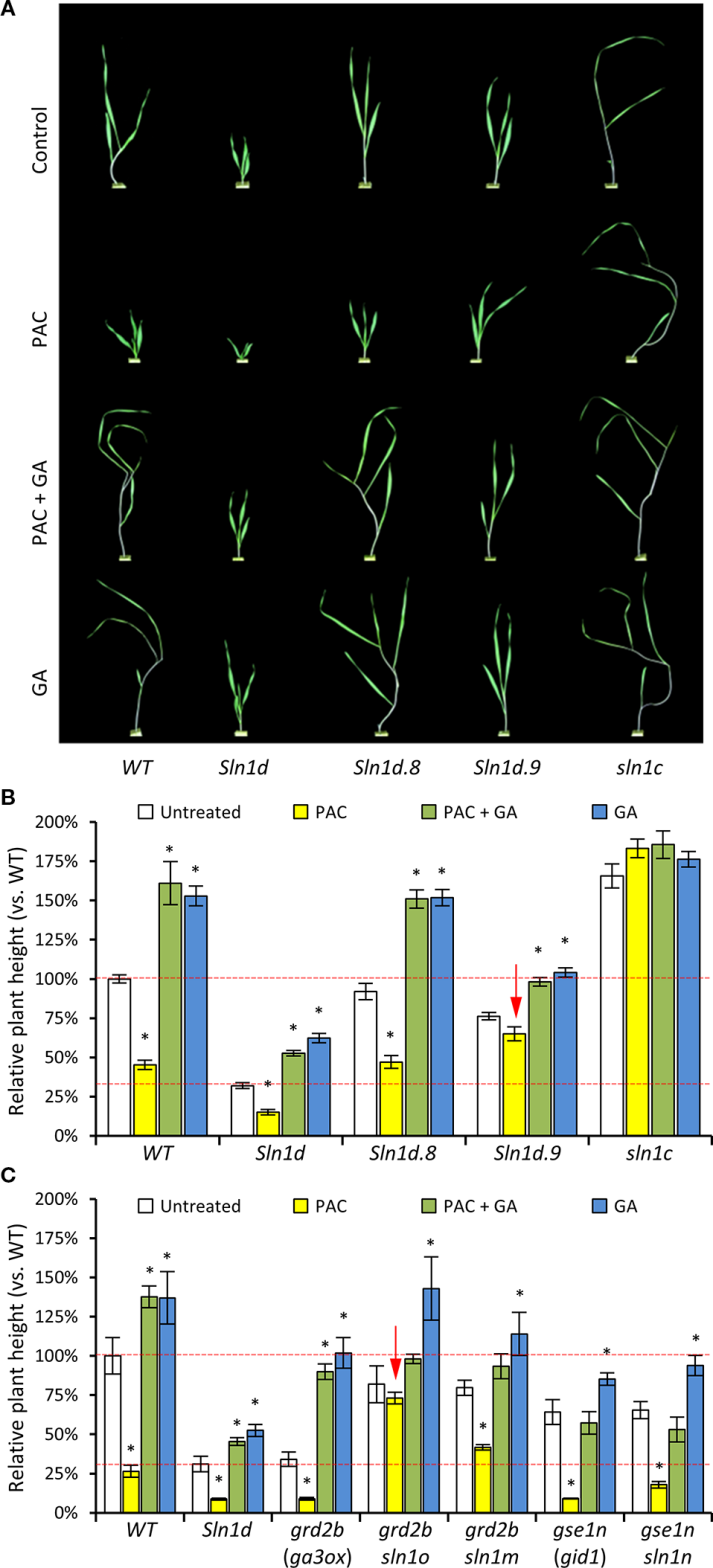
Gibberellin (GA) responsiveness and paclobutrazol resistance of different “*overgrowth*” *DELLA* alleles in barley. **(A)** Representative images of 2 week old (post-emergence) barley plants from the different GA/paclobutrazol treatments. **(B)** Plant height (linear length) of WT, GA-insensitive progenitor *sln1d*, two *overgrowth* alleles *sln1d.8* and *sln1d.9*, and loss-of-function *sln1c* in response to *in planta* changes in GA concentrations *via* application of 1x10^−5^M GA (GA) and 1x10^−6^M paclobutrazol (PAC). Red dashed lines indicate ideal plant height range. **(C)** Plant height in response to *in planta* changes to GA concentrations *via* applications of 1x10^−5^M GA (GA) and 1x10^−6^M paclobutrazol (PAC) of additional *sln1 overgrowth* alleles derived from GA deficient *ga3ox* and GA insensitive *gid1* mutant progenitor. Red arrows point out *overgrowth* alleles with the desired resistance to paclobutrazol. N = 10–15 plants for each treatment in both **(B**, **C)**. Asterisks above bars in **(B**, **C)** indicate statistical different between the treated *vs.* the untreated group (Student’s *t*-test p < 0.05). Error bars are S.E.M.

Two of the barley *DELLA overgrowth* alleles (*sln1d.8* and *slnd1.9*), which carry a secondary point-mutations that partially alleviates the severe dwarfing effect of *sln1d*, were assessed for their capacity to confer resistance to paclobutrazol at concentrations sufficient to cause severe dwarfing in both wild-type cereals and wild radish (**Figures 10A, B**). Growth of WT barley was substantially retarded in response to paclobutrazol, which was alleviated with co-application of GA. As expected, the overly “slender” loss-of-function *sln1c* allele, with its lack of the GA-regulated DELLA growth suppressing protein, was impervious to both increases and decreases in GA levels (**Figures 10A, B**). Plants carrying the excessively dwarfing *sln1d* allele showed a slight sensitivity to changes in GA levels, consistent with the *sln1d* mutant protein still being partially GA responsive (Willige et al., 2007; Chandler and Harding, 2013). Even so, the growth response of the dwarfed *sln1d* mutant to changes in GA levels is less than that of WT barley (**Figures 10A, B**). The secondary mutation in the *sln1d.8* allele restores untreated growth levels of *sln1d* back to WT (**Figure 10B**). It also reestablished a similar sensitivity to both GA and paclobutrazol, making *sln1d.8* unattractive as a GA-inhibitor resistant genotype. On the other hand the *sln1d.9* allele appears promising, as it partially restored untreated plant height to within the preferred semi-dwarfed range and dampened growth response to applied GA (**Figure 10B**). Most importantly, the *sln1d.9* plants were essentially resistant to paclobutrazol induced growth inhibition, retaining a level of growth similar to the untreated *sln1d.9* plants and remaining within the desired semi-dwarfed range (i.e., between WT and *sln1d* untreated lines; **Figure 10B**).

We also tested paclobutrazol and GA responses of the *sln1o*, *sln1m*, and *sln1n overgrowth* alleles. These *sln1 overgrowth* mutants were isolated from the dwarfed genetic backgrounds of the GA deficient *grd2b (barley GA3OX*) and GA insensitive *gse1n* (*barley GID1*—GA receptor) mutants, respectively. This means that these particular alleles have only a single mutation in the *sln1* gene and do not carry the primary dominant GA-insensitive dwarfing *sln1d* mutation present in *sln1d.8* and *sln1d.9* (Chandler and Harding, 2013) (**Figure 10C**). Plants carrying the *sln1m* or *sln1n* alleles are phenotypically similar to *sln1d.8* in that they respond to both GA and paclobutrazol (**Figure 10C**). Like the *sln1d.9* allele, the *sln1o* allele is resistant to paclobutrazol but responsive to added GA (**Figure 10C**). The *sln1o* does differs from *sln1d.9* in that it shows a far greater GA stimulated growth (**Figure 10C**). This is likely the outcome of *sln1o* being generated in a *ga3ox* background and therefore not co-carrying the GA-insensitive *sln1d* mutation and thus encoding for a DELLA protein that is still susceptible to GA-mediated degradation. Alleles such as *sln1o* that are resistant to decreases in GA (e.g., paclobutrazol treatment) but still responsive to elevated GA levels may provide unique modes of actions and effects on other GA controlled phenotypes such as grain size that may be more favorable than alleles with reduced sensitivity to both increases and decreases in GA (e.g., *sln1d.9*).

**Figure 11:**
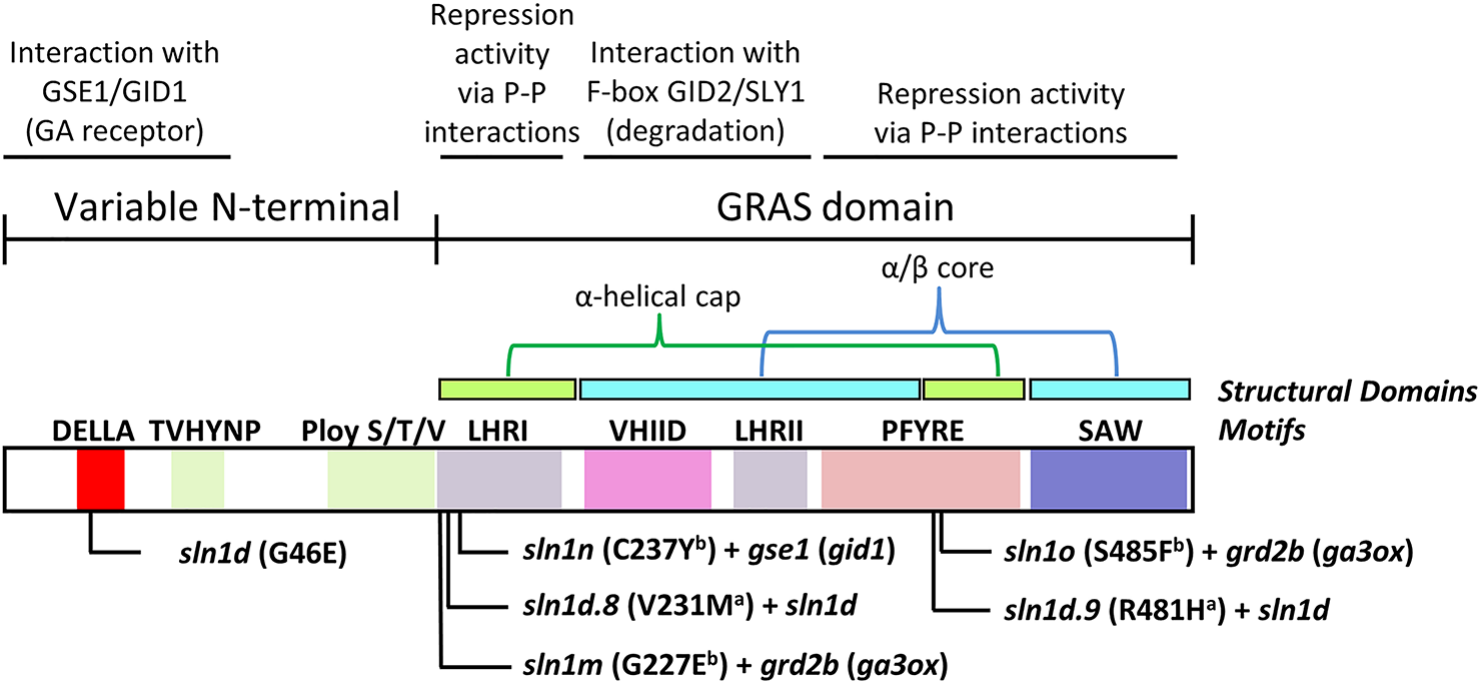
Graphical representation of the SLN1 protein, showing functional motifs, tertiary structural domains, and the *overgrowth* mutations. The primary *sln1d* mutation resides in the GID1 interacting DELLA domain of the GRAS-family variable N-terminal region. ^a^ *sln1d.8* and *sln1d.9* were generated in the *sln1d* background and therefore contain the original *sln1d* mutation in addition to the new point-mutation indicated. ^b^ *sln1m* and *sln1o* were generated in the *grd2b* (barley *ga3ox*) mutant background and *sln1n* was generated in the *gse1n* (barley *gid1*) mutant background, and so only carry a single point-mutation in *sln1* (i.e., no GA-insensitive *sln1d* mutation). The paclobutrazol resistant *sln1d.9* and *sln1o* alleles both reside in the PFYRE motif that helps form one-half of the α-helical cap structural domain. The other alleles occur in the LHRI motif that forms the other half of the α-helical cap. P-P, protein-protein interactions. Mutation information and locations obtained from (Chandler and Harding, 2013).

Interestingly, the *sln1 overgrowth* mutations providing paclobutrazol resistant (i.e., *Sln1d.9* and *sln1o*) occur within the PFYRE protein motif, which forms one-half of the α-helical cap structure (**Figure 11**). Whereas those *sln1 overgrowth* mutations found not to provide paclobutrazol resistant (i.e., *sln1d.8*, *sln1m* and *sln1n*), all reside within the LHRI motif forming the other half of α-helical cap structure (**Figure 11**).

## Discussion

### Targeting the Gibberellin Pathway Will Detrimentally Impact Traits That Make Wild Radish a Successful Weed

In this study we explored the possibility of pursuing gibberellin biosynthesis as a novel molecular target for controlling wild radish, and in doing so provide new insights into GA biology. By characterizing *ga3ox* mutants in *Arabidopsis*, a close taxonomic relative to wild radish, we established that even mild GA deficiencies can cause considerable reductions in growth and fecundity. The dwarfing and reduced seed set of *ga3ox* mutants is consistent with reports of decreased growth and reduced fecundity in other GA biosynthetic mutants (e.g., *ga20ox* and *ga2ox*) (Rieu et al., 2008a; Rieu et al., 2008b). In *ga20ox* and *ga2ox* mutants, the GA associated reductions in seed set are generally reported as abnormalities in male fertility, with defects in stamen filament elongation, tapetum development, pollen production, and pollen tube growth (Plackett et al., 2011; Plackett et al., 2012; Kay et al., 2013; Plackett et al., 2014). We found similar abnormalities with the *ga3ox* mutants, beginning with malformed anthers with reduced pollen quantities. The mature pollen that was produced was less viable, with impaired germination efficiency that could not be rescued by exogenous GA application. This implies that the abnormal mature pollen of *ga3ox* mutants are a result of defects arising earlier in anther/pollen development. This is consistent with the known roles of GA in tapetum and early pollen development, as determined from studies of other GA biosynthetic mutants (Plackett et al., 2011). Through reciprocal hand pollinations, we could also attribute the decrease in *ga3ox* fecundity to defects in female reproductive tissues. The deficiency occurred as a reduction in the fertilization rate of ovules, even when *3ox* mutant pistils were manually fertilized with wild-type pollen. Genetic studies on GA signaling loss-of-function *della* mutants have implicated GA in the proper development of female reproductive tissues (Fuentes et al., 2012). Double *gid1* mutants also show reduced seed set related to defects in maternal tissues (Gallego-Giraldo et al., 2014). Additionally, the dominant dwarfing gain-of-function *gai-1* (*GIBBERELLIC ACID INSENSITIVE*) DELLA mutant reduces seed set which is not related to pollen defects or seed abortion, and so likely female infertility (Swain et al., 2004). However, to the best of our knowledge, our observations are the first to report an explicit effect on female fertility rates attributed to a deficiency in GA biosynthesis. The *Arabidopsis ga20ox1 ga20ox2* double mutant produces fewer seed than wild type, but this is seemingly due to a reduction in ovule numbers as opposed to a decrease in ovule fertility rate (Rieu et al., 2008b; Plackett and Wilson, 2016). The inability of past studies to recognize an explicit female infertility phenotype in GA biosynthesis mutants, is likely a result of it not easily manifesting in other mild GA deficient mutants due to differences in tissue specific expression between GA biosynthesis genes and/or masking pleotropic effects. Ecotype differences may also play a role, as our *ga3ox* mutants and the *gai-1* mutant are in the L*er* (Landsberg *erecta*) background, whereas all the other mentioned GA mutants are in the Col (Columbia) background. There are indications that GA regulated maternal processes may differ between L*er* and Col (Swain et al., 2004). The female fertility defects would also unlikely be specifically discernible in severe GA deficient mutants because of their gross and more extensive floral morphological defects. Although severe *ga1* alleles are anecdotally known to be female sterile (Swain, personal communication).

Given the promising results in *Arabidopsis* of effecting traits key to making wild radish a successful weed species, we turned our attention to characterizing GA deficient phenotypes in wild radish itself. We found that chemical suppression of GA biosynthesis under controlled conditions could impair the growth rate and high seed production of wild radish, which are traits fundamental to its success as a weed. The slower growth and dwarfism of GA deficient wild radish should allow the wheat crop to establish and suppress the wild radish (Weiner et al., 2001; Eslami et al., 2006; Borger et al., 2010). GA deficiencies also had a direct impact on seed set through effecting GA regulated growth of reproductive tissues. The fertility defects seen in the mild GA deficient *Arabidopsis ga3ox* mutants translated to chemically induced GA deficient wild radish. This included a reduction in female fertility, which further supports a role for GA promoting female-specific aspects of fecundity. The anther and pollen defects, affecting male fertility, are likely to be more prominent in wild radish given its obligated out-crossing propensity (Pierson et al., 2013). The dwarfing of wild radish should also exacerbate the decrease in its overall seed production given the substantial reduction in flower number.

### Sufficient Differences Exist Between Wheat and Wild Radish GA3OX Proteins to Reasonably Expect That Preferential Gibberellin Inhibitors Can Be Found

Toward exploring the possibility of a wild radish specific GA inhibitor, we focused on the GA3OX proteins that catalyze the final step of GA_9_ to GA_4_ and are targets of a number of inhibitors. We cloned *RrGA3OX1a* and *RrGA3OX2a* and confirmed GA 3-oxidase activity *in planta* and more directly using a novel adaptation of the GID1-DELLA Y2H system. Next we used phylogenetic comparisons, protein sequence analysis and protein modeling to refine and evaluate if amino acid differences between wild radish and wheat GA3OXs occur at locations likely to define substrate specificity.

Phylogenetic and MEME analysis, identified a series of motifs conserved in sequence and position among the GAOX proteins. Several GAOX class-specific motifs were identified with a unique combination of four motifs characterizing the GA3OX proteins (motifs 20, 4, 14, and 18). Modeled tertiary structures revealed that the reaction cavities of RrGA3OX and TaGA3OX proteins consisted of a superior GA docking pocket with the narrow depths of the cavity housing the FE(II) and 2OG co-substrates. This configuration is consistent across a diverse set of 2ODD enzymes for which crystal structures complexed with bound substrates have been resolved (**Supplementary Figure S11**). By mapping the MEME motifs to the tertiary structures, we found that the motif set characterizing GA3OX proteins, contributes substantially to the formation of the GA binding pocket. This strongly implicates that the GA3OX-specific motifs, and their unique counterparts at equivalent locations in GA20OX and GA2OX proteins, have been recruited to define GA_n_ specificity and activity.

Other studies on GAOX and other 2ODD proteins, support the likely importance of these GA3OX motifs for specifying GA recognition and metabolism. Motif 14 overlaps the KLPWKETLS domain of GA20OXs, which is considered to define affinity for GA_12_ and GA_53_ (Xu et al., 1995; Sakamoto et al., 2004). Parts of both motifs 4 and 14 overlap a stretch of sequence characterized as being functionally divergent and under positive selection in GA3OX proteins (Huang et al., 2015). A number of studies have also observed the C-terminal tail (i.e., GA3OX motif 18), to exert important control over 2ODD enzyme activity. For instance, the C-terminal region overlapping motif 8 of GA20OXs (the positional homolog of GA3OX motif 18; **Figure 6C**), is involved in regulating GA_n_ specificity (Lange et al., 1997). Additionally, deletion within the equivalent C-terminal region in C_20_-GA2OXs, eliminates or greatly reduces enzyme activity (Lo et al., 2008). The C-terminal tail also influences specificity and activity in the non-GAOX 2ODDs of DEACETOXYCEPHALOSPORIN C SYNTHASE (DAOCS), ISOPENICILLIN N SYNTHASE (IPNS), and PROLYL HYDROXYLASE DOMAIN 2 (PHD2) (Lee et al., 2001; Chowdhury et al., 2016; McNeill et al., 2017).

Contained within the motifs forming the reaction cavity and the residues lining the GA pocket, we identified 17 possible GA interacting residues, which are the stronger of the candidates for defining GA_n_ variant specificity. Ten of these residues occur within the GA3OX characteristic motifs. Consistent with their likely functional importance, many of these residues were highly conserved in GAOX proteins, with conservation sometimes restricted to a specific class, reconcilable with their differential specificity for GA_n_ variants. Notable variations were also observed between GA3OXs from wheat, wild radish, and their orthologs, at several of the 17 possible GA interacting positions. These identified residues serve as strong candidates for further structural, mutagenesis, and dynamic protein modeling studies, which will help establish the molecular criteria conferring GA_n_ variant recognition and directing site-specific GA modifications.

Admittedly, other residues lining the GA docking pocket may influence GA_n_ specificity. This may occur either through steric changes altering positions of interacting residues, or *via* extended bonding networks. For instance, distinctions between RrGA3OX and TaGA3OX at position 229 [Ala(A) *vs.* Ile(I)] may be of significance given that a Ala(A) > Thr(T) substitution at this site in the *ga3ox le-1* mutant allele of pea, causes a 100-fold increased substrate affinity for the precursor molecule GA_9_ over the wild type *LE* allele (Martin et al., 1997) (**Figure 7**). Whereas, a substitution of His(H) > Tyr(Y) at position 276 reduces the GA 3-oxidase efficiency of the pea *le-3* mutant allele (Martin et al., 1997) and a Cys(C) > Tyr(Y) at position 219 eliminates GA 3-oxidase activity in the *Arabidopsis 3ox1-1* mutant (Chiang et al., 1995). Even residues with similar properties and only minor steric changes [e.g., Leu(L) to Ile(I), Asp(D) to Glu(E), or Lys(K) to Arg(R)] can be poorly tolerated in the 2ODD reaction cavity, leading to altered activity rates or changes in substrate specificity (Gebhardt et al., 2007; McNeill et al., 2017). This is also a factor specifically for GA recognition, where minor steric substitutions in the GA binding pocket of the GID1 GA receptor, lead to increased affinity for either GA_34_ or GA_9_ at the expense of GA_4_ (Shimada et al., 2008). We observed that in wheat, wild radish, and their close orthologs, the motifs forming the GA binding pocket have a number of amino acid polymorphisms that are highly conserved to either the monocot or dicot GA3OXs, suggesting a functional relevance to these deviations. Such numerous and biochemically diverse polymorphism will almost certainly culminate in reaction cavities with different capacity for GA_n_ recognition and modification, between monocots and dicot Brassicales GA3OXs. This notion is supported by several reported observations; while AtGA3OXs are strict 3-oxidases, TaGA3OX-B2, and OsGA3OX1 principally act as 3-oxidases but also have minor GA 2-oxidase, 2,3-desaturase, and even 13-hydroxylase activities, indicating interactive networks capable of rotating the docked GA substrate (Itoh et al., 2001; Appleford et al., 2006); GA3OXs from wheat, rice, and maize can recognize and convert GA_5_ to GA_3_, while those from dicot *Arabidopsis* and pea cannot (Martin et al., 1997; Williams et al., 1998; Itoh et al., 2001; Appleford et al., 2006; Chen et al., 2014); although GA_9_ is slightly preferred over GA_20_ by TaGA3OX-B2, the differences is much less than the 10-fold preference for GA_9_ over GA_20_ by AtGA3OX1 (Williams et al., 1998; Appleford et al., 2006). By extension, it would also be reasonable to expect that the diversity in the identified key candidate motifs and residues between RrGA3OX and TaGA3OX proteins, would allow for a dicot specific inhibitor targeting wild radish over a monocot cereal such as wheat to be possible. This conclusion is supported by the 16,17-dihydro-GA group of GA biosynthesis inhibitors, some of which show the opposite of our desired specificity by preferentially inhibiting monocot over dicot GA3OX activity (Evans et al., 1994; Foster et al., 1997; King et al., 2004; Rademacher, 2018).

### Toward Discovering Species-Specific GA3OX Inhibitors

For several decades, chemical screens have identified inhibitors of bioactive GA synthesis in plants. More recently, with the increasing availability of chemical libraries and omic information on GA related enzymes, target directed screens have successfully identified a suite of chemicals functioning as bioactive GA mimics or GA receptor inhibitors, to highly specific inhibitors of GA2OXs (Jiang and Asami, 2018). The GA3OX GID1-DELLA yeast system we established to determine the GA 3-oxidase capability of RrGA3OX1a, could be used to screen for inhibitory molecules that discriminate between dicot and monocot GA3OX enzymes. In support of this, chemical screens using the basic GID1-DELLA version of this yeast system have identified inhibitors of the GID1-DELLA interaction (Yoon et al., 2013), and has been used to evaluate possible off-target inhibition of GID1-DELLA interaction by new derivatives of 16,17-dihydro-GA_5_ (Tian et al., 2020). The GA3OX GID1-DELLA yeast system is semi-quantitative as the GID1-DELLA dependent yeast growth rates are sensitive to GA_4_ concentrations (**Supplementary Figure S12**). Although not used in this study, the interaction can also be monitored colormetrically using the LacZ reporter, providing a more sensitive quantification of the GID1-DELLA interaction or inhibition thereof. Fluorescent (e.g., GFP) or bioluminescent (e.g., luciferases) reporters could be engineered into the system, to offer dynamic continuous real-time monitoring. Our version of the system could be broadened to utilize species specific GID1 proteins and potentially even include the upstream GA20OX proteins, expanding the number of targets and increasing the probability of isolating a species-specific GA inhibitor. The insight into species-specific differences of possible GA interacting residues, could help tailor specificity through structure-activity relationship (SAR) studies, much like inhibitors of auxin, abscisic acid, and ethylene biosynthesis/signaling have been refined (Jiang and Asami, 2018).

### Making Cereals Resistant to Gibberellin Inhibitors as an Alternative Approach

The *sln1* and *rht-B1 overgrowth* alleles present an alternative to developing a species specific GA inhibitor. This second approach is likely more practical as it does not require the development of new chemicals and approval for use in an agricultural setting. The *overgrowth* alleles are already being integrated into cereal breeding programs because of their semi-dwarfing growth, improved grain characteristics, and potential benefits toward several other important agronomic traits regulated by DELLAs (Chandler and Harding, 2013; Derkx et al., 2017; Serrano-Mislata et al., 2017; Thomas, 2017; Van De Velde et al., 2017a; Van De Velde et al., 2017b; Mo et al., 2018; Moriconi et al., 2019). Some of these *overgrowth* alleles will also have the added benefit of conferring resistance to GA inhibitors, extending the potential agricultural applications of these *overgrowth* alleles.

The second site *sln1 overgrowth* mutations rescue the *sln1d*, *grdb2*, and *gse1n* dwarf phenotypes by either decreasing the abundance, or more likely for the alleles we examined, by making the SLN1 DELLA protein less effective at growth suppression. The extent of this feature would raise and set the threshold levels of DELLA units required to exert an actual developmental effect, with DELLA concentration being influenced by the resistant/sensitivity of the sln1 protein to GA-mediate degradation. Therefore the different responses of the *sln1 overgrowth* alleles to paclobutrazol and GA treatments, are an outcome of an intricate interplay between GA concentrations, resistance of sln1 to GA-mediated degradation, and the constraint on triggering growth suppression processes imposed by the secondary *overgrowth* mutation. In accordance almost all of the identified s*ln1* and *rht-B1 overgrowth* mutations, reside within domains involved in mediating protein-protein interactions responsible for the intrinsic repression activity of DELLA proteins (Hirano et al., 2010; Hirano et al., 2012; Chandler and Harding, 2013; Derkx et al., 2017; Van De Velde et al., 2017b). The wheat *overgrowth* mutations frequently occur in the LHRI and PFYRE domains, and only occur in these domains for barley (Derkx et al., 2017). Their distribution occurs throughout LHRI, but reside only in the tail end of PFYRE domain. Significantly, the tertiary structure of DELLA proteins consists of an α-helical cap sitting atop of the α/β core, with the entire LHRI domain and the tail end of the PFYRE domain each constituting one-half of the α-helical cap tertiary structure (Hakoshima, 2018). Of the alleles we tested, those found not to increase paclobutrazol resistant (i.e. *Sln1d.8*, *sln1m*, and *sln1n*), all had secondary *overgrowth* mutations within the LHRI domain, whereas the mutations increasing paclobutrazol resistant (i.e. *sln1d.9* and *sln1o*) occur within the α-helical cap portion of the PFYRE domain (**Figure 11**) (Chandler and Harding, 2013). The α-helical cap is thought to mediate SLN1 head-to-head homo- and heterodimerization, and known to facilitate interacts with other proteins (Van De Velde et al., 2017b; Hakoshima, 2018). Dozens of direct DELLA binding proteins have been reported in various plant species, from transcriptional activators to chromatin remodelers, and even co-chaperones, with the outcome of DELLA interaction triggering growth suppression (Van De Velde et al., 2017b). Although the binding modes are currently unknown, it is possible that the two structural halves of the α-helical cap mediate independent interactions or their respective importance varies with different partners. This would explain the distinct growth responses to changes in GA concentrations, and the resistance level to paclobutrazol, between the LHRI and PFYRE residing *overgrowth* mutations. This differing response to paclobutrazol is consistent with overgrowth allele-specific differences for other GA-related traits such as α-amylase production and grain size (Chandler and Harding, 2013). Of the 27 *Rht-B1* suppressor alleles that exist in wheat, four of these reside within the PFYRE domain (*Rht-B1c.15* to *Rht-B1c.18*) (Chandler and Harding, 2013; Derkx et al., 2017). *Rht-B1c.15* is a strong candidate as a semi-dwarfed paclobutrazol resistance wheat variety as it carries the same Arg(R) to His(H) substitution as the barley *sln1d.9*.

Using PFYRE domain *overgrowth* alleles in wheat could provide additional yield improving benefits by allowing the use of GA inhibitors as a part of an integrated weed management program. With the growing acceptance of CRISPR gene editing for commercial crop applications, GA inhibitor resistant *overgrowth* alleles could be duplicated to benefit other cropping systems.

## Conclusion

Wild radish is a formidable weed of Australian cropping systems, in particular burdening the wheat industry with costs of control and reductions in yield (Reeves et al., 1981; Cheam and Code, 1995; Jones et al., 2005; Llewellyn et al., 2016). With the continued emergence of resistance to commonly used herbicides, new innovative molecular approaches are needed to control wild radish weed populations that could be used in conjunction with improved weed management farming practices (Cheam and Cheam, 2008; Walsh et al., 2017) (http://www.grdc.com.au). In this study we explored the possibility of manipulating gibberellin control over growth and fertility as a possible translatable application for managing wild radish weed populations in cropping systems. Our results suggest that targeting the GA pathway could be a viable inclusion in wild radish management programs that warrants further investigation. In drawing this conclusion, we provided new insights into GA regulated reproductive biology and molecular characteristics of GA biosynthesis and DELLA signaling proteins. We also developed and identified tools that would benefit the future development of this work. The yeast system could be used to screen and evaluate chemical inhibitors, while the paclobutrazol resistant *overgrowth* barley and equivalent wheat genotypes, provide an opportunity to test the strategy of using GA inhibitors to control wild radish.

## Experimental Procedures

### Plant Growth Conditions

Wild radish plants were from two natural populations in Western Australia; AL (Albany; 34°49′S, 117°58′E) and PG4 (NE of Perth; 31°24′S, 116°19′E). All *Arabidopsis* lines are in a Landsberg *erecta* background, unless otherwise stated. The barley lines are all of the Himalaya variety. *Arabidopsis*, wild radish and barley were grown at 22–24°C under 18/6 h light/dark photoperiod (100–150 µmol m^−2^ s^−1^ for *Arabidopsis* and wild radish; 200 µmol m^−2^ s^−1^ for barley). Soil grown plants (*Arabidopsis* and wild radish) were sown in Debco Seed Raising Mix and watered with nutrient solution. Hydroponically grown wild radish and barley were sown into GroWool (Horticultural Systems) and watered with nutrient solution. Where applicable, GA and/or paclobutrazol treatment was administered at 2 weeks after sowing for wild radish, and shortly after coleoptile emergence for barley; subset of plants were subjected to a single dose of either paclobutrazol (1x10^−6^M or 1x10^−7^M) and/or GA_3_ (1x10^−5^ M) introduced into the nutrient solution. For GA/PAC application of wild radish main stems, healthy primary bolts were excised from the plant and then a secondary cut was performed ~2.5 cm above the primary cut to aid in the removal of air emboli and restore water uptake and positive water balance. The stems were inserted into foil wrapped 50 ml flacon tubes through small holes in the lids to minimize evaporation of the water containing nutrient solution supplemented with GA/PAC. Cutting were placed in standard growth conditions above and grown for 2 weeks.

### Plant Strains

*Arabidopsis Atga3ox2-1* mutants were transformed by infection with *Agrobacterium tumefaciens* using the floral dip method (Clough and Bent, 1998). Independent T_1_ *Atga3ox2-1 35S:RrGA3OX1a* transgenic plants were isolated using BASTA spray application on soil sown seed. Seed from 10 “taller” appearing T1 plants were collected. The T2 generation was examined for complementation of the *Atga3ox2-1* dwarf phenotype. For all transgenic lines scored, the presence of the transgene and the homozygosity of the *Atga3ox2-1* mutant allele was confirmed by PCR (for primers see **Supplementary Table S7**). For semi-quantitative assessment of transgene expression, RNA from developing rosette leaves was extracted using the QIAGEN RNeasy Plant Mini Kit, treated with DNaseI (Invitrogen) as per manufactures instructions, and used with the QIAGEN One-Step RT-PCR kit; RT-PCR primers for *RrGA3OX1a* and *RrGA3OX2a* can be found in **Supplementary Table S7**. The

*Arabidopsis GA3OX* and *DELLA* mutants used in this study are all previously reported, well-studied, mutants; *Atga3ox1-1* (Chiang et al., 1995); *Atga3ox1-2* (Chiang et al., 1995); *Atga3ox2-1* (Mitchum et al., 2006)*; Atrgl2-5* (Lee et al., 2002). All mutants are in the Landsberg *erecta* background with the exception of *Atga3ox2-1* (Columbia accession) that was crossed into the L*er* background (ensuring *erecta* co-segregation) to generate the *ga3ox1-2 ga3ox2-1* double mutant.

### Scoring of Seed Set

Dried siliques on the primary bolt from within floral positions 5 to 15 were scored and fecundity reported as seed set per silique; no. of seed/no. of (seed + unfertilized ovules). The production of a seed was determined by either counting seed directly or in instances where the seed had abscised, by counting the funiculi which are distinguishable from those of unfertilized ovules.

### Pollen Assays

Assays were performed as described in (Kay et al., 2013). Pollen counts: estimated from one healthy anther at the point of dehiscence per flower from X individual flowers (X = sample size stipulated in figure legends). Pollen viability: pollen grains were stained with fluorescein diacetate and viewed under near ultraviolet light (350–400 nm) with viable pollen fluorescing yellow-green. *in vitro* pollen germination was performed as described in (Fan et al., 2001), with pollen considered viable if producing an elongated pollen tube.

### Cloning of *RrGA3OX* and Other *GA3OX* Genes

Total RNA was extracted from wild radish (population AL) anthers using QIAGEN RNeasy Plant Mini Kit and treated with DNaseI (Invitrogen) as per manufactures instructions. Partial wild radish GA3OX sequences (internal cDNA sequence) were isolated using QIAGEN OneStep RT-PCR Kit and degenerate primers designed from a region within *GA3OX* conserved across *Arabidopsis* and a number of other Brassicaceae (**Supplementary Table S7**). Fragments were cleaned using QIAGEN PCR purification kit and cloned into Promega pGEM™-T vector and sequenced. The 5′ and 3′ ends of *RrGA3OX1a* and *RrGA3OX2a* were isolated using Clontech RACE Kit. Sequences were submitted to GenBank *RrGA3OX1a* (KP271966.1) and *RrGA3OX2a* (KP271967.1). *35S:RrGA3OX1a* was created by amplifying coding region using primers incorporating restriction sites (**Supplementary Table S7**) compatible for insertion into the 35S promoter containing vector pART7. *35S:RrGA3OX1a* cassette was cleaved and inserted into pMLBART binary transformation vector using flanking *Not*I sites and transferred into *Agrobacterium tumefaciens* strain AGL1 as described in (Groszmann et al., 2008). Yeast expression GA3OX1 constructs were cloned using primers incorporating restriction sites (**Supplementary Table S7**) compatible for insertion into yeast expression vector pYX212. The constructs were transformed into the AH109 yeast strain carrying the GID1-SLR1 yeast two-hybrid system (Ueguchi-Tanaka et al., 2007) according to the protocol set out in the Clontech Matchmaker manual. Yeast cultures were grown as described in the Clontech Matchmaker manual with the addition of either histidine, GA_4_, or GA_9_ as described in main text.

### Yeast Liquid Cultures and Analysis of Gibberellin 3-oxidase Activity

Yeast was grown in SD dropout medium minus appropriate amino acids to select for and maintain *GID1*, *DELLA*, and *GA3OX* expression vectors. One milliliter overnight starting cultures were spun down, washed, and resuspended in phosphate buffer (pH 7), with ~10 µl of the resuspension used to inoculate a 1 ml experimental culture to achieve an initial starting OD_600_ ~0.1. The growth medium was supplemented with either histidine, GA_4_, or GA_9_ as described in main text. Where required, the optical density (OD_600_) of yeast cultures were measured using an Eppendorf BioPhotometer^®^ Plus compact UV/Vis photometer. Secondary confirmation of the presence of GA_4_ in yeast supernatant was performed using GC-MS as described in (Ross et al., 1995).

### Protein Modeling

Protein alignments were generated using MUSCLE (Edgar, 2004). Secondary structures were predicted using both PSIPRED (http://bioinf.cs.ucl.ac.uk/psipred/) (Buchan et al., 2013; Buchan and Jones, 2019) and SWISS-MODEL (https://swissmodel.expasy.org/) (Waterhouse et al., 2018). SWISS-MODEL using default parameters was used to homology model the tertiary structures of the RrGA3OX and TaGA3OX enzymes using the best templates according to global quality estimation (GMQE) and qualitative model energy analysis scores (QMEAN) (Waterhouse et al., 2018); these being 2-ODD enzymes *Arabidopsis* ANTHOCYANIDIN SYNTHASE (AtANS; PDBID: 1GP4, 1GP5, and 1GP6) (Wilmouth et al., 2002) and *P. somniferum* (Poppy) THEBAINE 6-O-DEMETHYLASE (T6ODM; PDBID: 5o7y and 5o9w) (Kluza et al., 2018). Full protein sequence: RrGA3OX1a/1gp4.1: GMQE 0.63, QMEAN −2.48; RrGA3OX1a/5o7y.1: GMQE 0.66, QMEAN −3.32; TaGA3OX2-1/1gp4.1: GMQE 0.62, QMEAN −2.7; TaGA3OX2-1/5o7y.1: GMQE 0.61, QMEAN −2.26. Truncated protein sequence (matching sequence length of crystal structures): RrGA3OX1a/1gp4.1: GMQE 0.7, QMEAN −2.07; RrGA3OX1a/5o7y.1: GMQE 0.72, QMEAN −2.98; TaGA3OX2-1/1gp4.1: GMQE 0.7, QMEAN −2.72; TaGA3OX2-1/5o7y.1: GMQE 0.68, QMEAN −2.60. Protein illustrations were generated through either NGL viewer (Rose and Hildebrand, 2015) within the SWISS-MODEL workspace, or through UCSF-Chimera (https://www.cgl.ucsf.edu/chimera/) (Pettersen et al., 2004). Motif-based sequence analysis was performed using Multiple EM for Motif Elicitation (MEME) and Motif Alignment and Search Tool (MAST) from the MEME suite toolkit (version 5.1.0: http://meme-suite.org/index.html) (Bailey et al., 2015), invoking default settings with maximum number of motifs = 20, minimum motif length = 6, maximum motif length = 50.

### Protein Sequences and Phylogenies

*RrGA3OX1a* and *RrGA3OX2a* CDS and protein sequences have been deposited into National Center for Biotechnology Information (NCBI) database; accession numbers (KP271966) and (KP271967) respectively. GA3OX, GA20OX, and GA2OX sequences across the angiosperms were obtained partially from (Huang et al., 2015) with additional sequences identified with BLASTp at NCBI using *Arabidopsis* and rice GAOX sequences as queries. *R. raphanistrum* genomic and predicted protein sequences were mined from RadishDB: http://radish.plantbiology.msu.edu/index.php?title=RadishDB. *R. sativus* genomic and predicted protein sequences were mined from RadishGD: http://radish-genome.org/. All protein sequences used in this study can be found in **Supplementary Tables S1**–**S5**. Protein sequences were aligned using MUSCLE sequence aligner and phylogenetic trees generated using MEGA7 (http://www.megasoftware.net/) (Kumar et al., 2016). Phylogenies were constructed using neighbor joining method invoking partial deletions and bootstrap 500x options. The evolutionary distances were computed using the Poisson correction method and reported as the frequency of amino acid substitutions per site. The analysis involved 516 amino acid sequences. There were a total of 1,116 positions examined in the final dataset.

## Data Availability Statement

The datasets generated for this study can be found in the Genbank RrGA3OX1a (KP271966), Genbank RrGA3OX2a (KP271967).

## Author Contributions

MG and SS conceived project. MG performed the research. JR performed gas chromatography–mass spectrometry (GC-MS) analysis. PC provided *overgrowth* mutant resources. MG wrote manuscript. SS critically reviewed manuscript.

## Funding

The work was supported by the CRC for Australian Weed Management and the Australian Research Council Centre of Excellence for Translational Photosynthesis (CE140100015).

## Conflict of Interest

The authors declare that the research was conducted in the absence of any commercial or financial relationships that could be construed as a potential conflict of interest.
