# Supplementary material for: Manipulating Gibberellin Control Over Growth and Fertility as a Possible Target for Managing Wild Radish Weed Populations in Cropping Systems
Source: Front Plant Sci. 2020 Mar 19;11:190. doi: 10.3389/fpls.2020.00190 (PMC7096587; doi:10.3389/fpls.2020.00190)
Supplement: Supplementary file 2 [file DataSheet_2.zip › Supplementary Figures.PDF]

Supplementary Material

Manipulating gibberellin control over growth and fertility as a possible target for managing wild radish weed populations in cropping systems

Michael Groszmann, Peter M Chandler, John J Ross, Steve M Swain

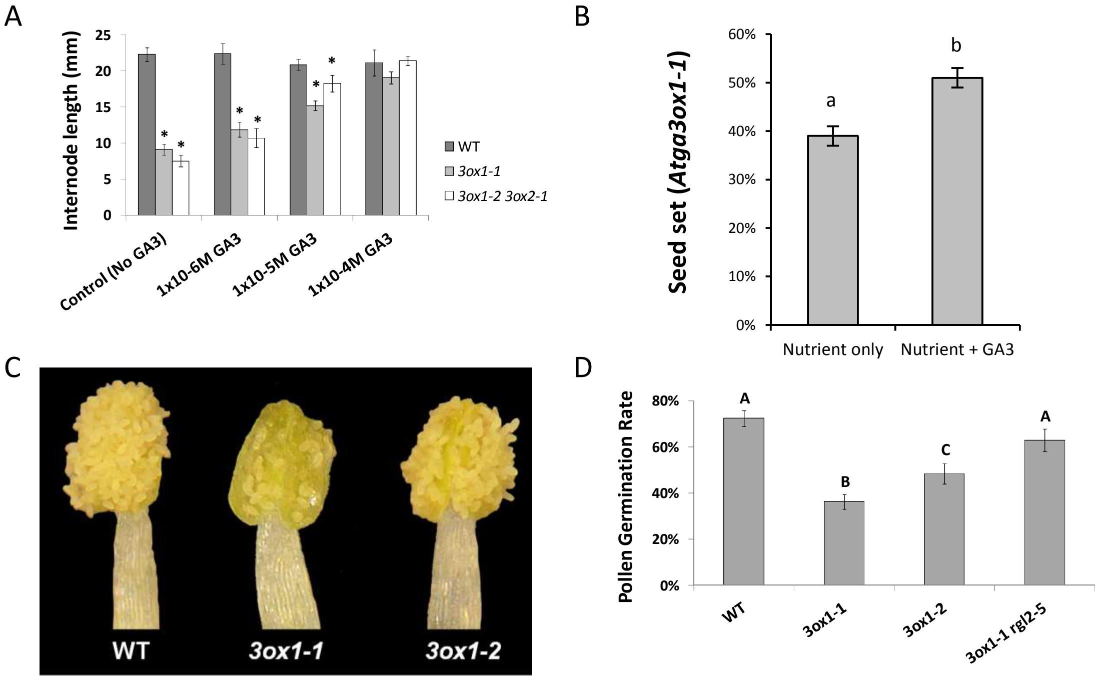

Supplementary Figure S1. Additional characterization of growth and fertility defects of Arabidopsis ga3ox mutants (A) Rescue of the short stature of Arabidopsis *ga3ox1* mutants via exogenous application of GA. Asterisks = significant difference between *Atga3ox* mutants and *wild type* (*WT*); Student's *t*-test $p < 0.05$. **(B)** Improved fecundity of *Atga3ox1*-1 mutant treated with GA (via root drench). **(C)** Representative anthers produced by *wild type* (*WT*), *Atga3ox1-1* and *Atga3ox1-2* plants showing abnormal anther development and poor pollen production in the *ga3ox1* mutants compared to *WT*. **(D)** Pollen viability as assessed by *in vitro* pollen germination rate. Patterns correlate with pollen viability assayed using fluorescein diacetate (see Figure 1F in main text). Letters above bars (B) and (D) represent pairwise statistical comparison between categories. Categories marked with a given letter are statistically different from categories marked with another letter (Student's *t*-test $p < 0.05$). All error bars are S.E.M.

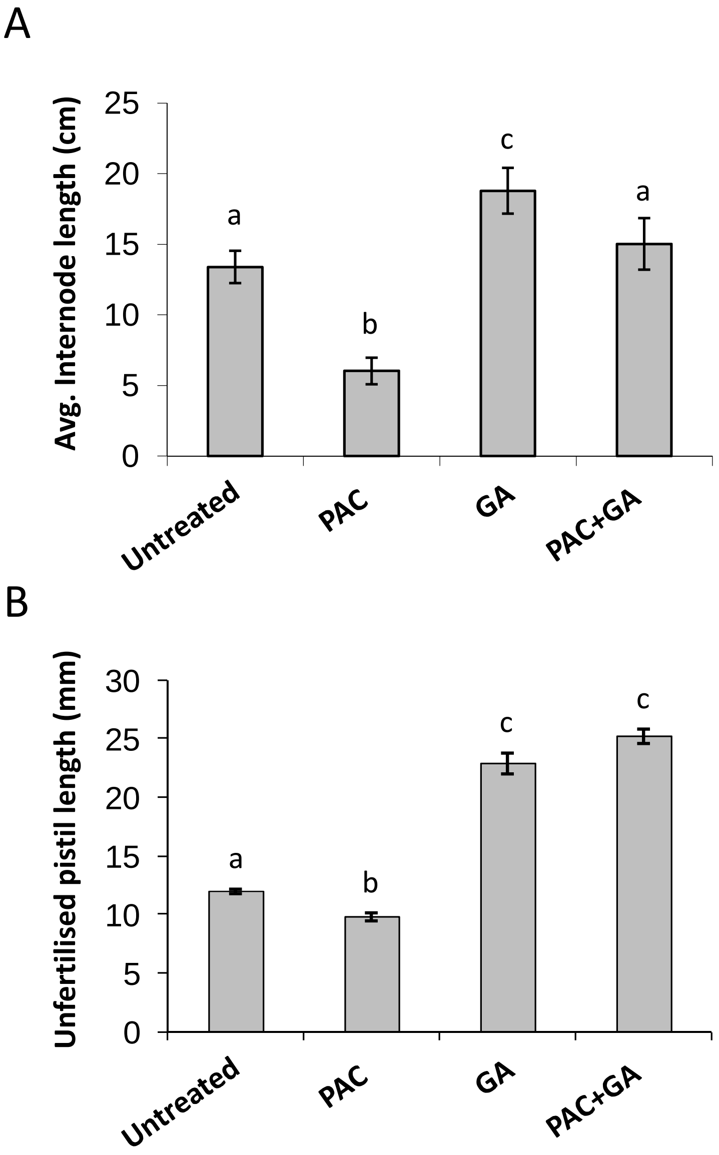

Supplementary Figure S2. Wild radish main shoot cuttings treated with GA and Paclobutrazol. (A) Internode elongation of wild radish main shoot cuttings placed into nutrient solution supplement with GA ($1x10^{-5}$ GA_3) and or paclobutrazol (PAC) ($1x10^{-5}$). **(B)** Length of unfertilised pistils produced on the main shoot cutting from (A). Letters above bars in (A) and (B) represent pairwise statistical comparison between categories. Categories marked with a given letter are statistically different from categories marked with another letter (Student's *t*-test $p < 0.05$). Error bars are S.E.M.

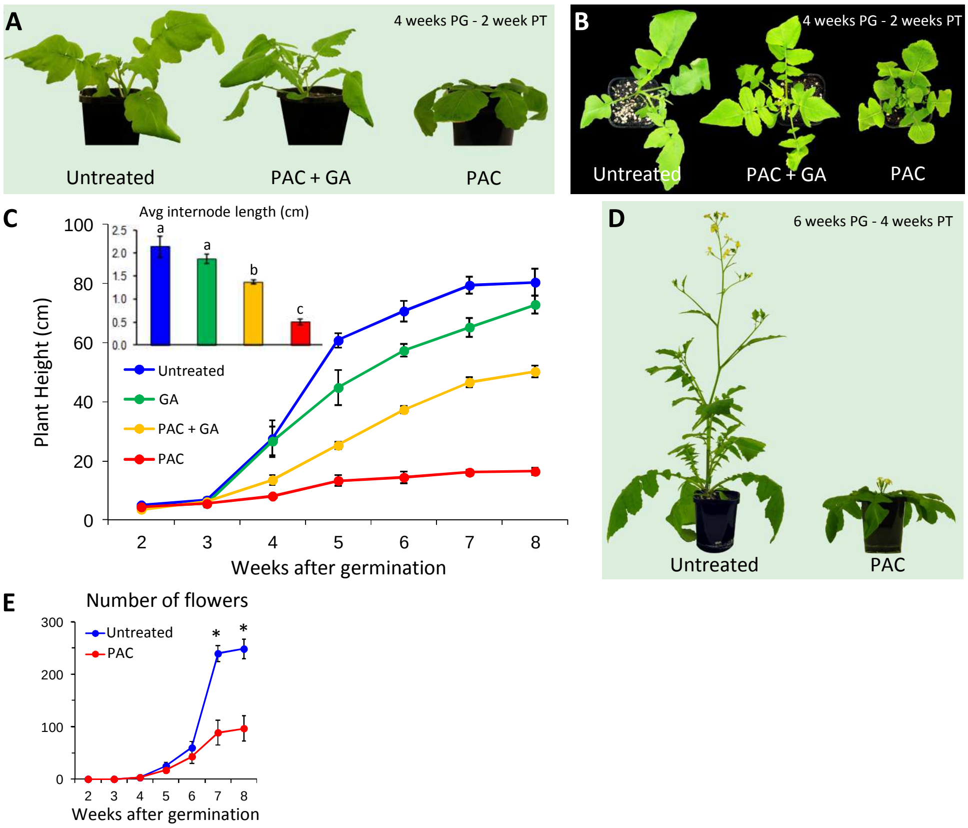

Supplementary Figure S3. Growth and fertility defects induced by paclobutrazol treatment of soil grown wild radish. (A-B) Representative images of wild radish showing the impact on growth induced by paclobutrazol (PAC) treatment; paclobutrazol ($1x10^{-6}M$) and/or GA_3 ($1x10^{-5}M$). PG: post-germination, PT: post-treatment. **(C)** Growth, represented as plant height, over a 8 week period. Insert; bar chart showing average internode length at 8 weeks. Lettering denotes statistical differences between categories (Student's *t*-test $p < 0.05$). **(D)** Representative soil grown plants at 6 weeks PG (4 weeks PT). **(E)** PAC treatment reduces the number of flowers (reproductive units) in wild radish. Asterisks denotes statistical differences between categories (Student's *t*-test $p < 0.05$). N in (C) and (E) = 8 plants for each treatment. All error bars are S.E.M.

A

>Rr3OX1a [Raphanus raphanistrum] full length GA 3-OXIDASE1a gene (CDS) (GenBank: KP271966.1)
ATGCCTACAATGTTAACAGATGTCTTTAGAGGCCATCCCATTCACCTTCCACACTTTCACCAACCTGACTTAACATCCCTCAGTAATCTCCCGGATTCTTACACGTGGACCTCCCAAG
ACGATCCTCTCTTCACCGTTGCTCCTCCTCCGACCGCCGGTGAAAACATCCCTCTCATTGACCTGAACAACCCCGACGCGGCCAACCAAATCGGCCGTGCATGTAGAACTTGGGGTGC
GTTCCAGATCGTAAACCATGGCGTGCCTCTGGAACTTCTCCAAGGCATCGAGTTTCTCACCGGTAATCTTTTTAGGCTACCTGTCCAGCGCAAGCTTAAGGCGGCTCGGTCAGAGACA
GGTTTCTCTGGCTACGGCGTTGCACGTATCTCATCTTTCTTTAATAAGAAAATGTGGTCCGAAGGTTTCACCATCACCGGTTCCCCTCTCAATGATTTCCGTAAACTTTGGCCCCAAC
TTCACCTTGACTACTGCGACATCGTTGAACAGTACGAGGAACAAATGCAAAAGTTGTCATCGAAGTTGATGTGGCTATCACTGAAGTCACTTGGCGTCAGCGAAGAAGACATTAAATG
GGCCAGGGGCAGTTCAGATTTTAACTGGGCCCAATCCGCCCTCCAGCTAAACCACTATCCGGTTTGTCCTGAACCCGACCGGGCTATGGGTCTAGCCCCCCATACCGATTCGACCCTC
TTAACTATACTCTACCAGAACAATACCGCCGGTCTCCAAGTATTTCGTGATGATCTTAGTTGGGTCACCGTGCCACCGGTTCCAGGCTCCCTCGTGGTAAATGTTGGTGATCTTTTCC
ACATTCTATCCAACGGTTTGTTTAAAAGTGTGATTCACCGTGCTCGGGTTAACCAAACCAGATCTCGGTTATCCGTAGCTTTCCTCTGGGGTCCACAGTCTGATATCAAGATATCACC
TGTACTAAAACTGGTTAGGCCTGATGAATCGCCTTTATACCGATCGGTCACATGGACAGGGTATCTCCGAACAAAAGCAACTCACTTCAATAAAGCTCTTTCAATGATCAGAAATCAC
AGACAGGGATAA

>RrGA3OX1a full length GA 3-OXIDASE1a protein (GenBank: AKH87502.1)
MPTMLTDVFRGHPIHLPHFHQPDLTSLSNLPDSYTWTSQDDPLFTVAPPPTAGENIPLIDLNNPDAANQIGRACRTWGAFQIVNHGVPLELLQGIEFLTGNLFRLPVQRKLKAARSET
GFSGYGVARISSFFNKKMWSEGFTITGSPLNDFRKLWPQLHLDYCDIVEQYEEQMQKLSSKLMWLSLKSLGVSEEDIKWARGSSDFNWAQSALQLNHYPVCPEPDRAMGLAPHTDSTL
LTILYQNNTAGLQVFRDDLSWVTVPPVPGSLVVNVGDLFHILSNGLFKSVIHRARVNQTRSRLSVAFLWGPQSDIKISPVLKLVRPDESPLYRSVTWTGYLRTKATHFNKALSMIRNH
RQG*

B

>RrGA3OX2a [Raphanus raphanistrum] full length GA 3-OXIDASE2a gene (CDS) (GenBank: KP271967.1)
ATGAGTTCAACGTTGAGCGATGTGTTCATATCACATCCCATTCACATCCCGTTCTCAAACTTACCCGACTTCACATCCCTTCGTCACCTCCCAGACTCTTACACGTGGACCCCCAAAG
ACGATCTCCTCTTCTCCGCCTCCGCGTCTGACGAATCTCTACCATTCATCGACCTCTCCGATCCCCACGTGGCCTCTCGTGTTGGCCATGCTTGCACCACGTGGGGGGCATTCCAGAT
CACAAACCACGGCGTGCCCTCACGGCTTCTCGACGACATGGAGTTCCTCACGGGAAGCCTTTTCCGGCTTCCCGTCCATCGGAAGCTCAATGCTGCTCGACGTGAGGATGGCATATCC
GGCTACGGCGTTGCTCGTATTGCTTCATTCTTCAACAAGCAAATGTGGTCCGAAGGTTTCACAGTTGTTGGTTCCCCACTTGACGATTTCCACAAACTCTGGCCCGTACATCACCTCA
AATACTGTGAAATTATCCAAGAGTATGAAGAGCATATGCAAAAGTTGGCAGCGAAGCTGATGTGGCTGGCATTAGGTTCACTTGGAGTTGAGGAAAAAGACATTGAATGGGCCAGTCC
CGGTTCAGACTTTCGAGGAGCACAAGCAGCTATCCAACTCAACCACTATCCGATATGTCCAGAACCGGACCGAGCAATGGGCCTCCCAGCACATACCGACTCAACTCTCATGACCATA
CTGTACCAGAACAACACCGCCGGTCTCCAGGTTTACCGCGATGACGTGGGATGGGTCACTGTGCCACCTGTCCCTGGCTCACTAGTAGTCAACGTCGGTGACTTACTCCACATTTTGA
CCAACGGAATGTTCCCGAGCGTGCTTCACCGAGCCAGGGTTAACCACCTCCAATCTCGCTTCTCTATGGCTTACCTGTGGGGTCCACCTTCTGATCTGATGATCTCTCCGCTTCCAAA
ACTCGTTGATCCTCTCCATCCTCCTCTTTACCCATCTCTTTCTTGGAAGCAGTACCTTGCAACCAAAGCTACTCATTTTAACCAGTCTCTTTCCTTTATTAGGAACTATCGGTCTTCA
GACCAAATCTCTTGA

>RrGA3OX2a full length GA 3-OXIDASE2a protein (GenBank: AKH87503.1)
MSSTLSDVFISHPIHIPFSNLPDFTSLRHLPDSYTWTPKDDLLFSASASDESLPFIDLSDPHVASRVGHACTTWGAFQITNHGVPSRLLDDMEFLTGSLFRLPVHRKLNAARREDGIS
GYGVARIASFFNKQMWSEGFTVVGSPLDDFHKLWPVHHLKYCEIIQEYEEHMQKLAAKLMWLALGSLGVEEKDIEWASPGSDFRGAQAAIQLNHYPICPEPDRAMGLPAHTDSTLMTI
LYQNNTAGLQVYRDDVGWVTVPPVPGSLVVNVGDLLHILTNGMFPSVLHRARVNHLQSRFSMAYLWGPPSDLMISPLPKLVDPLHPPLYPSLSWKQYLATKATHFNQSLSFIRNYRSS
DQIS*

C

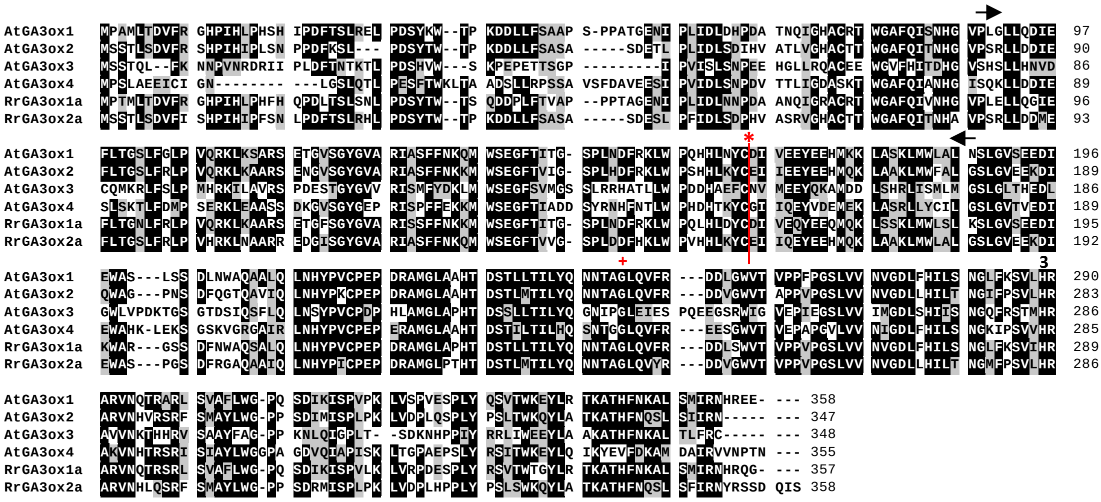

D

	AtGA3OX1	AtGA3OX2	AtGA3OX3	AtGA3OX4	RrGA3OX1a
**AtGA3OX1**	-	-	-	-	-
**AtGA3OX2**	72.90%	-	-	-	-
**AtGA3OX3**	40.50%	41.70%	-	-	-
**AtGA3OX4**	53.20%	51.60%	39.40%	-	-
**RrGA3OX1a**	84.90%	69.10%	42.30%	52.10%	-
**RrGA3OX2a**	70.70%	85.10%	41.60%	50.40%	66.20%

Supplementary Figure S4. Wild Radish GA3-OX sequences. **(A-B).** Nucleotide coding and protein sequences of *RrGA3OX1a* and *RrGA3OX2a*. **(C)** ClustalW protein alignment of the four Arabidopsis AtGA3OX proteins and the two wild radish RrGA3OX proteins. Black shaded residues are identical. Grey shading denotes related/similar residues between the sequences (60% threshold for shading). The red asterisks and vertical line depict the location of the first intron in all of the *AtGA3OX* genes and is contained in the region amplified by the two internal primer sets (black arrows) which detect the presence of the intron in both *RrGA3OX1a* and *RrGA3OX2a* (Figure S5). The additional symbol (+) denote the location of a second intron present only in *AtGA3OX3*. **(D)** Pair-wise comparisons of sequence homology between the different GA3-OX proteins from Arabidopsis and wild radish.

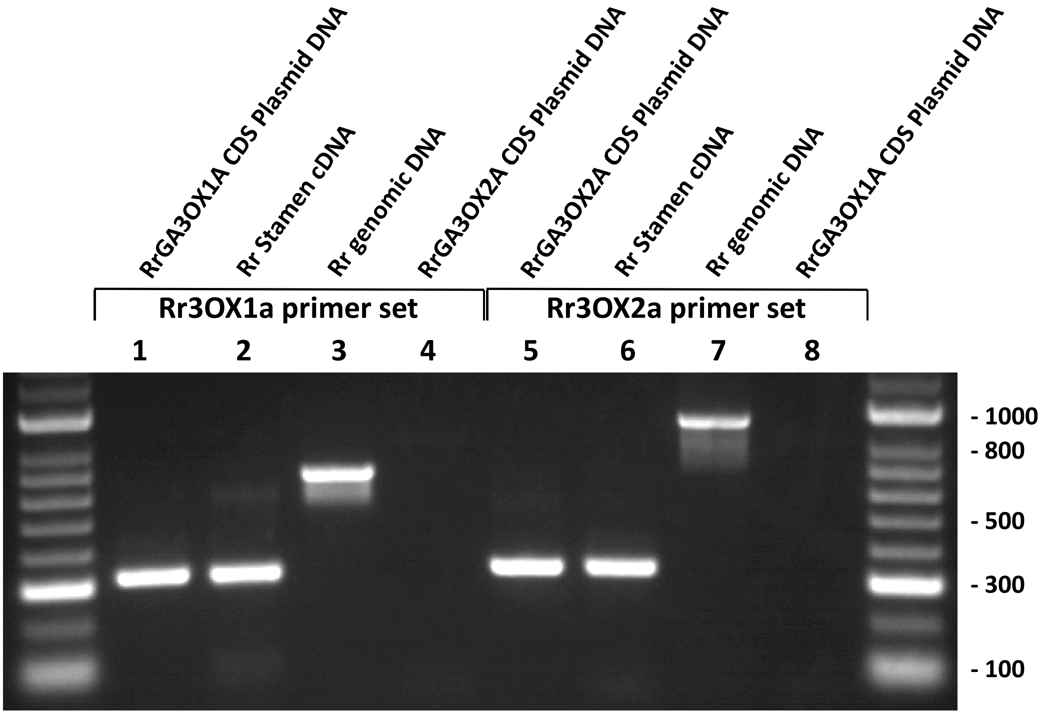

Supplementary Figure S5. Specificity of *RrGA3OX* primer used for RT-PCR experiments. Lanes 1-4: *RrGA3OX1A* specific primers. **Lanes 5-8:** *RrGA3OX2A* specific primers. **Lanes 1 and 5** indicate the expected PCR product size obtained if template is cDNA sequence. **Lanes 2 and 6** show the product sizes derived from cDNA which matches the expected sizes in the preceding lanes. **Lanes 3 and 7** indicate the product sizes if DNA contamination was present in RT-PCR results (i.e. intron sequence flanked by primers increases product length). **Lanes 4 and 8** shows primer specificity to their target gene and do not amplify the alternative *RrGA3OX* gene.

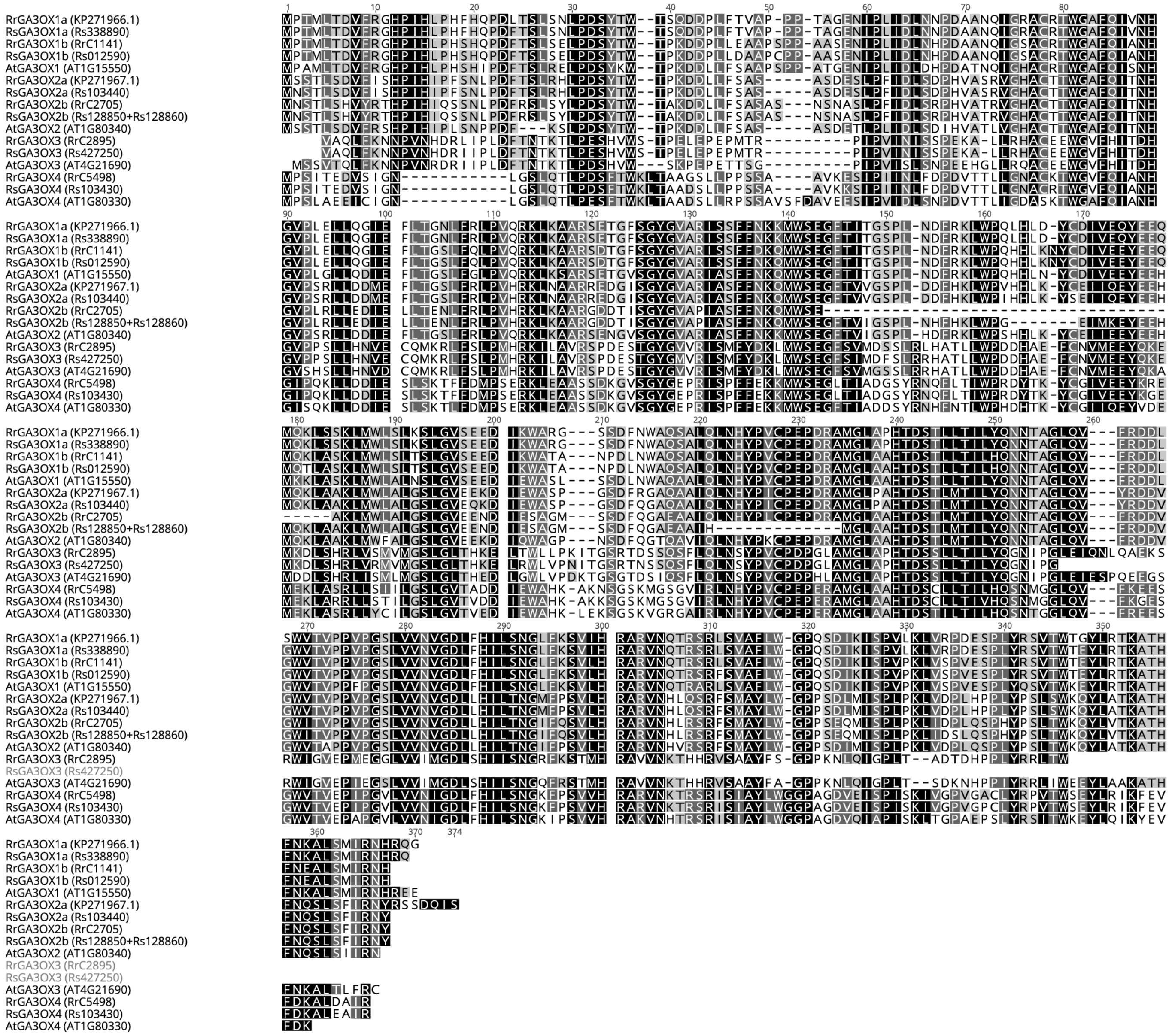

Supplementary Figure S6. Alignment of wild radish (Rr), *Raphanus sativus* (Rs), and Arabidopsis (At) GA3OX proteins. MUSCLE protein sequence alignment; shading denote amino acids with similar properties (based on ≥1 Blosum62 matrix score) and their prevalence across the 16 sequences (Black 100%; Dark grey 80-100%; Light grey 60-80%; white < 60%). GenBank (RrGA3OX1a and RrGA3OX2a), RadishDB (other Rr), RadishGB (Rs), and TAIR (At) gene/protein identifiers in parentheses adjacent to gene names. Due to an apparent lack of sequencing coverage only partial sequences were obtained for RrGA3OX2b, RsGA3OX2b and RsGA3OX3.

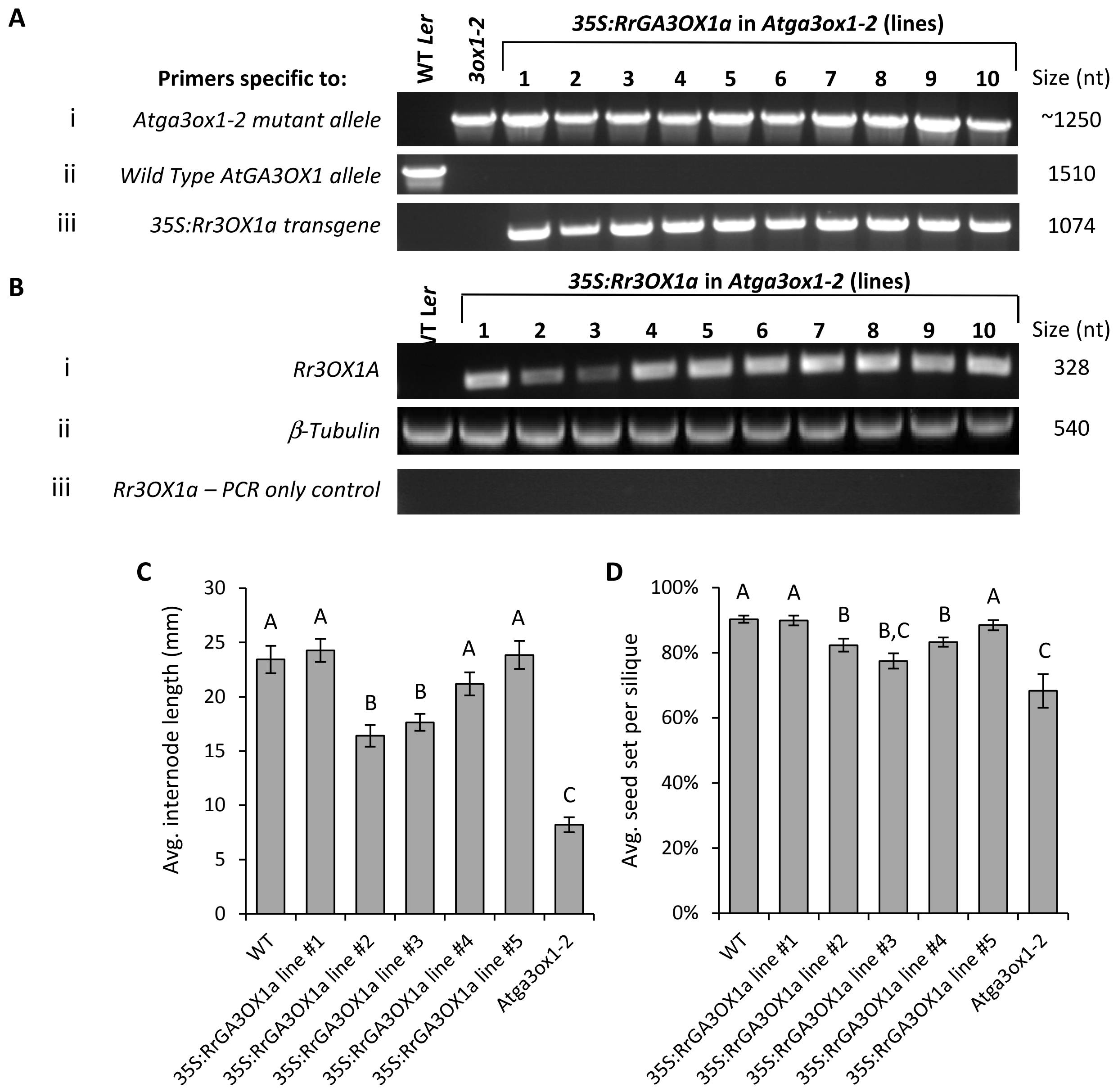

Supplementary Figure S7. Genotyping, expression and complementation assessment of *Atga3ox1-2* transgenic lines harboring the wild radish *RrGA3OX1a* CDS driven by the *35S* Cauliflower Mosaic Virus promoter. (A) Genotyping of T_2 *Atga3ox1-2* complemented lines confirmed that all lines tested were homozygous for the *Atga3ox1-2* mutant allele, while also carrying the *35S:RrGA3OX1a* transgene. **(Ai)** Detection of *Atga3ox1-2* mutant allele using a forward primer specific to the 5′ end of *AtGA3OX1* and a reverse primer specific to the T-DNA insert located in the intron of *Atga3ox1-2*. **(Aii)** Detection of *AtGA3OX1* wild type allele, using primers specific to the 5′ and 3′ ends of *AtGA3OX1*. **(Aiii)** Detection of *35S:RrGA3OX1a* transgene, using primers specific to the 5′ and 3′ end of the *RrGA3OX1a* gene. **(B)** Semi-quantitative RT-PCR analysis, detecting expression of the *35S:Rr3OX1a* transgene in the various complemented lines. **(Bi)** Detection of the *35S:RrGA3OX1a* transgene mRNA. **(Bii)** Tubulin loading control. **(Biii)** PCR only control (i.e. no RT step), showing below detectable level of DNA contamination of the RNA samples. **(C)** Average internode length quantifying the improved primary bolt elongation of independent *35S:RrGA3OX1a Atga3ox1-2* transgenic lines. (D) Average seed set per silique of independent *35S:RrGA3OX1a Atga3ox1-2* transgenic lines. Letters above bars in (C) and (D) represent a pairwise statistical comparison between categories. Categories marked with a given letter are statistically different from categories marked with another letter (Student's *t*-test $p < 0.05$). Error bars are S.E.M.

Supplementary Figure S8. Phylogenetic characterization of GA-OXIDASE proteins. Expanded version of the phylogeny depicted in Figure 6A.

482 GAOX and 34 related non-GAOX 2ODD protein sequences from across 37 angiosperm species. The non-GA3OX 2ODDs represent 2ODD enzymes that function in the biosynthesis or deactivation of flavonoids, jasmonic acid, salicylic acid, strigolactones, or auxin. The phylogeny is separated into five major clades, four of which represent the GA20OX, GA3OX, C_{19}-GA2OX and C_{20}-GA2OX classes of the GA-OXIDASE (GAOX) family. The GAOX sequences clearly segregate from the non-GAOX 2ODD representative group (green clade), indicating they are a distinct group of 2ODD proteins. GAOX functionality was confirmed by cross-matching against reported biochemical analysis, which confirmed the expected specific GAOX activity (i.e. GA20OX, GA3OX or GA2OX GA modifications) for numerous members from across the phylogeny (solid square preceding the sequence name; Supplementary Table S6).

Monocot (red clades) and dicot species (blue clades) are present in each of the GAOX classes but cluster separately from each other, suggesting significant independent diversification between monocot and dicot GAOX proteins. Substantial intra-order diversification is also apparent.

Sequences in bold are those indicated on the compressed version of the tree in Figure 6A.

Figure too large for PDF; See separate TIFF file

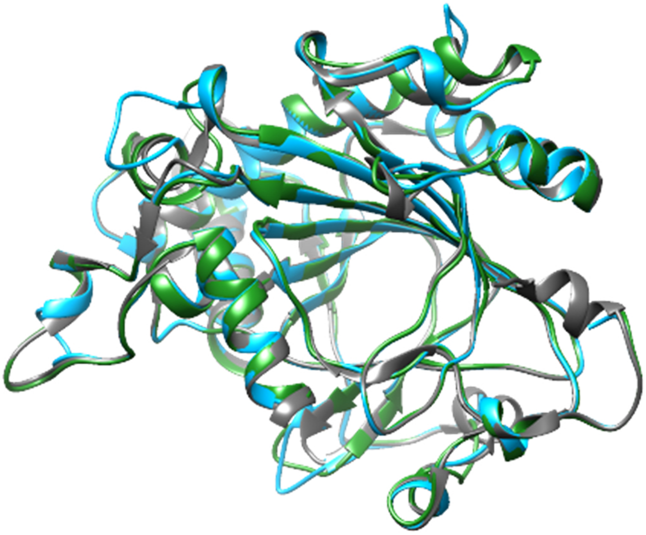

ASN 1gp4 (grey) template overlapped with RrGA3OX1a (Dark green) and TaGA3OX2-1 (cyan)

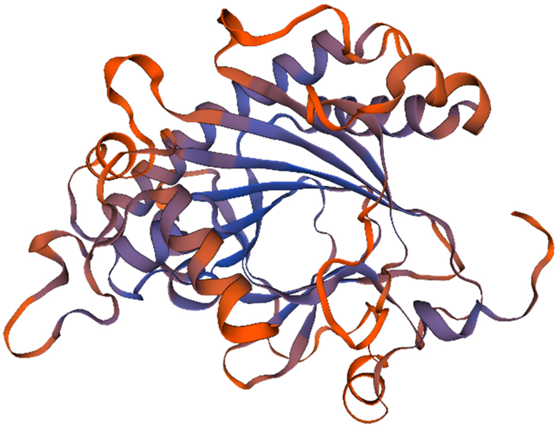

RrGA3OX1a modelled on ASN 1gp4

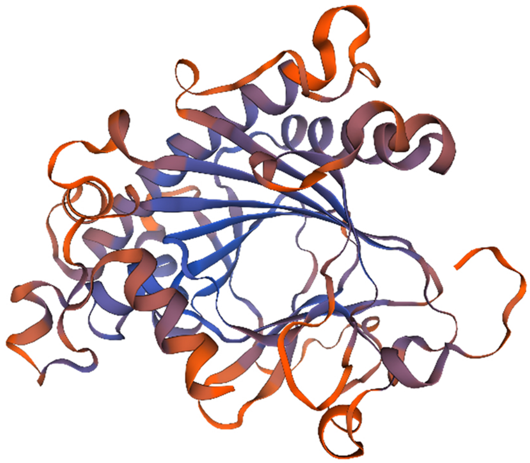

TaGA3OX2-1 modelled on ASN 1gp4

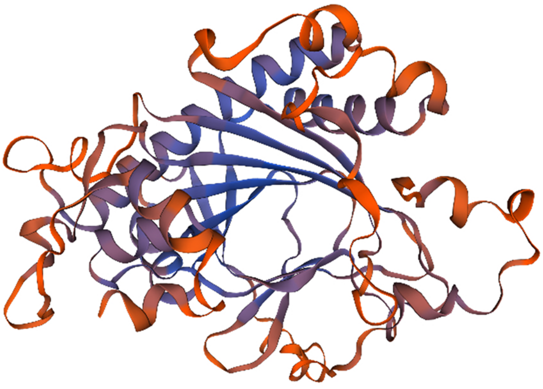

RrGA3OX1a modelled on T6ODM 5o7y

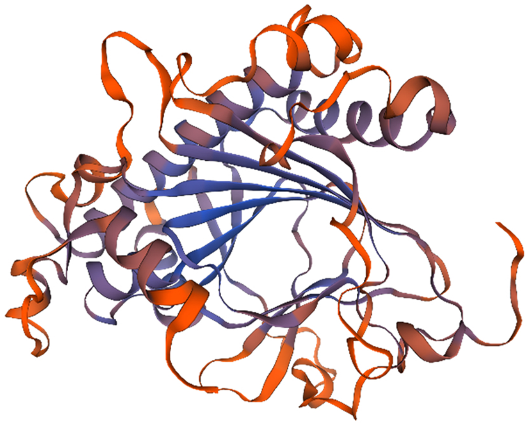

TaGA3OX2-1 modelled on T6ODM 5o7y

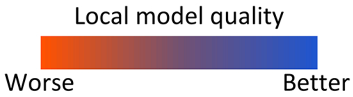

Supplementary Figure S9. SWISS-MODEL generated tertiary structures of the RrGA3OX and TaGA3OX. Homology model outputs using the resolved crystal structures of 2-ODD enzymes; Arabidopsis ANTHOCYANIDIN SYNTHASE (ANS; PDBID: 1GP4) (Wilmouth et al., 2002) and Papaver somniferum (Poppy) THEBAINE 6-O-DEMETHYLASE (T6ODM; PDBID: 5o7y) (Kluza et al., 2018) as templates. Local model quality is depicted on the tertiary structures by a colour gradient as measured according to all-atom lDDT scores.

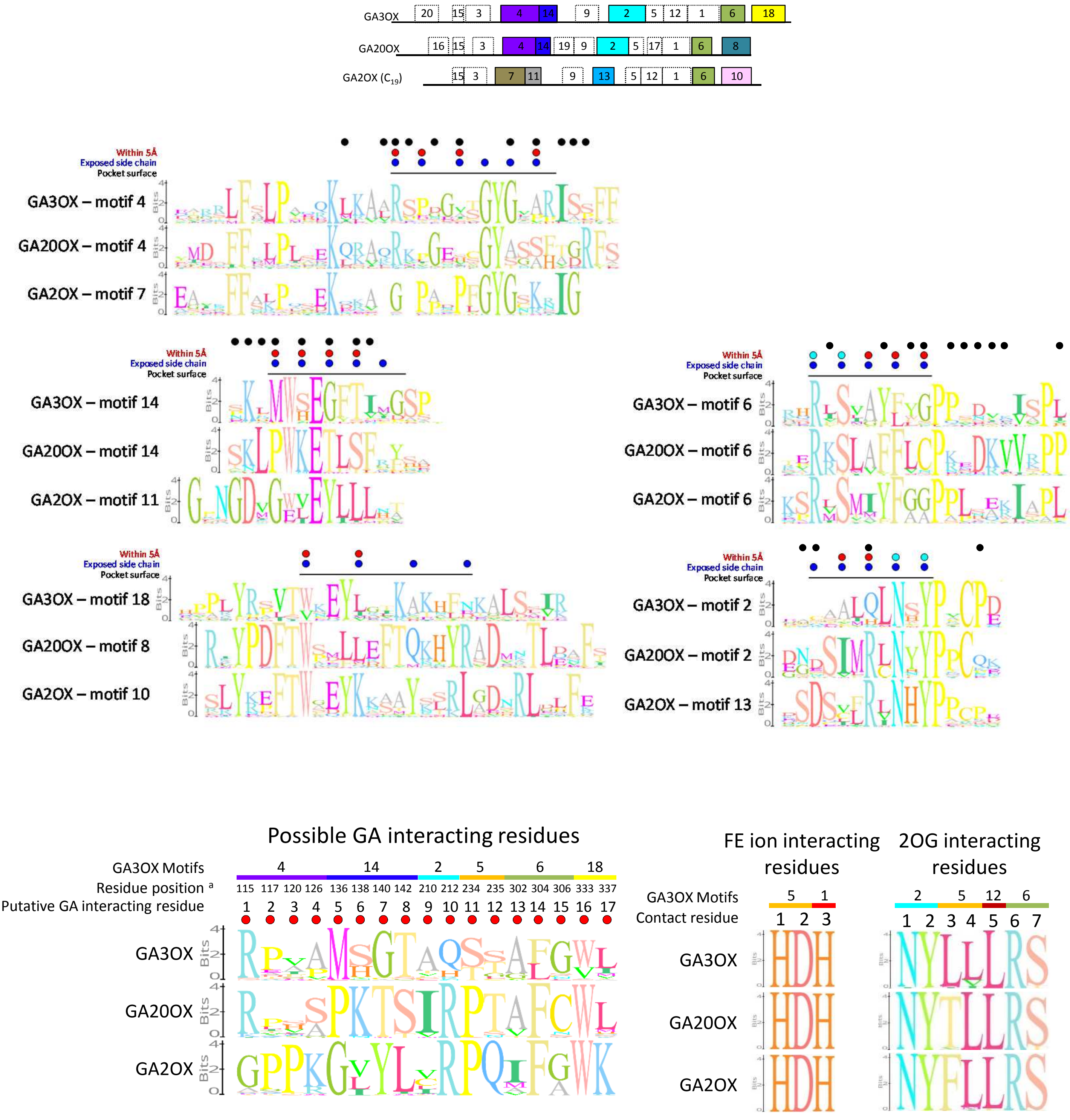

Supplementary Figure S10. Sequence logo alignments comparing the GAOX protein sequence motifs that directly form the GA binding pocket and residues involved in binding the FE ion and 2OG co-substrate.

The overall height of the residue logo stack indicates the sequence conservation at that position, whereas the height of the given residue reflects the relative frequency of the corresponding amino acid. Dots above the sequences: Blue - pocket lining residues with reactive side chains orientated inwards towards the substrate, Red - same as blue marked residues, except within 5Å of docked GA molecule, Black dots indicate notable conserved polymorphic residues involving substitutions of amino acids with distinctly different chemical properties. Black solid horizontal line: Residues of the motif that line the interior surface of the GA docking pocket. GAOX proteins are those used to build phylogeny in Figure 6A. N = 128 GA3OX, 155 GA20OX, 192 GA2OX. For sequences, see Supplementary Tables S2-S4. For their phylogenetic relationships, see Supplementary Figure 8. [a]Residue position in relation to the RrGA3OX1a sequence in Figure 7. Residues at the positions involved in interacting with the FE(II) ion and the 2OG co-substrate are essentially invariable between the classes, except for contact residue 3 of the 2OG interacting residues. This almost full conservation is consistent with these residues being involved in the core catalytic function of the GAOX proteins. The GA3OX motifs that form the GA reaction pocket share a number of highly conserved residues with their positional counterparts in GA2OX and GA20OX. These are likely core structural positions and residues essential for organizing the GAOX reaction cavity and generic GA_n interactions. The positionally homologous motifs also exhibit highly conserved polymorphisms, often involving amino acids with pronounced differing biochemical properties, which differentiate the three GAOX classes. Several of these conserved polymorphisms involve possible GA interacting residues, and are therefore strong candidates accounting for the specific recognition events differentiating the GAOX classes. The polymorphic residues show some interesting patterns of conservation. For example, the distinctly different configurations of the highly conserved Arg(R) residues at contact positions 1 and 10. Arginine is a complex amino acid able to form ionic interactions and up to 7 H-bond, in addition to standard van der Waals interactions. The positive charge of Arg(R) allows interaction with negatively-charged non-protein atoms, such as the carboxyl and hydroxyl groups of GA molecules. These differ in location on the GA ring structures between biologically active and inactive variants of GA (Figure 6B). Also interesting, are the highly conserved polymorphic residues at contact positions 7 and 8; especially the non-conservative exchanges in GA2OX to highly hydrophobic Tyr(Y) and Leu(L), the former being aromatic and capable of π-interactions. These occur in motif 14 and overlap the region occupied by the KLPWKETLS sequence of GA20OXs, thought to specify binding of GA_{12} and GA_{53} (Sakamoto et al., 2004, Xu et al., 1995).

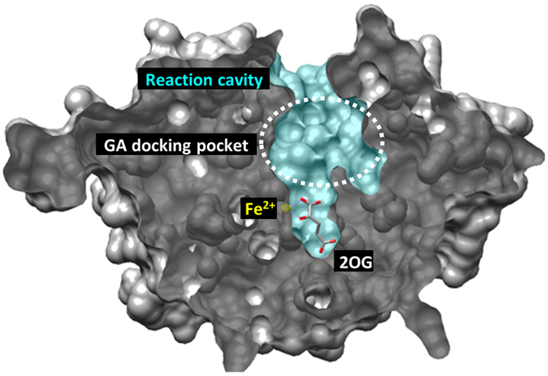

Modelled RrGA3OX1a

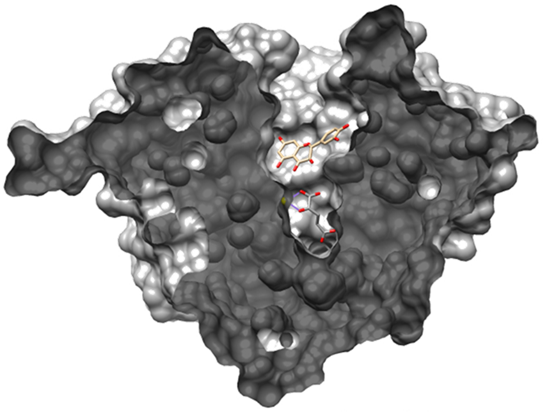

ANTHOCYANIDIN SYNTHASE (ASN) (PDB: 1gp5) with dihydroquercetin substrate (beige)

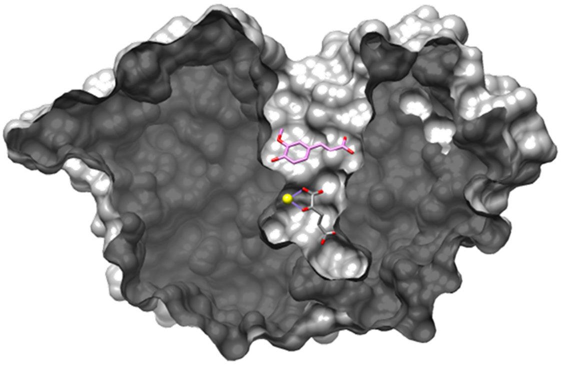

FERULOYL-CoA 6′-HYDROXYLASE1 (F6′H1) (PDB: 4xae) with Ferulic Acid substrate (purple)

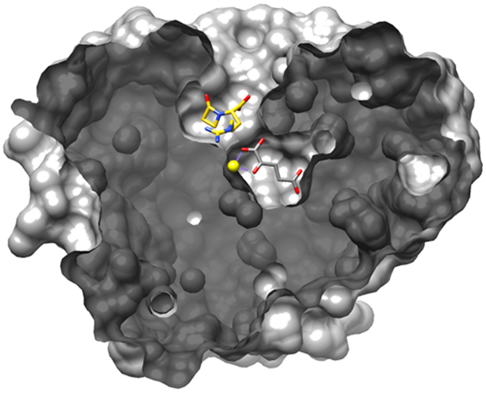

CLAVAMINATE SYNTHASE (CAS) (PDB: 1gvg) with PCX (Deoxyguanidinoproclavaminic acid) substrate (yellow)

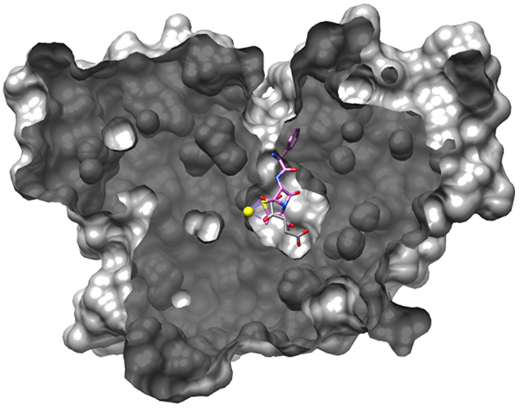

DEACETOXYCEPHALOSPORIN C SYNTHASE (DAOCS) (PDB: 1unb) with Ampicillin substrate (pink)

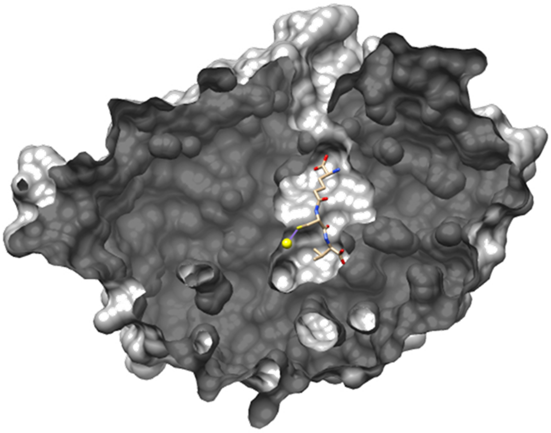

ISOPENICILLIN N SYNTHASE (IPNS) (PDB: 1bk0) with ACV substrate (beige)

Supplementary Figure S11. Configurations of the reaction cavities of several well studied 2ODD enzymes with resolved crystal structures. Images depict surface rendering of the tertiary structure followed by a longitudinal section to reveal the buried reaction cavity of several well studied 2ODD proteins. In each instance the FE(II) ion (yellow sphere) and 2OG co-substrate (grey) are located in the depths of the cavity. The main substrate docks into a pocket superior to the co-substrates. The orientation of the main substrate to the co-substrates determining the exact location of the chemical modification. Note that IPNS does not require the 2OG co-substrate, but the FE(II) ion is still situated in the depths of the cavity.

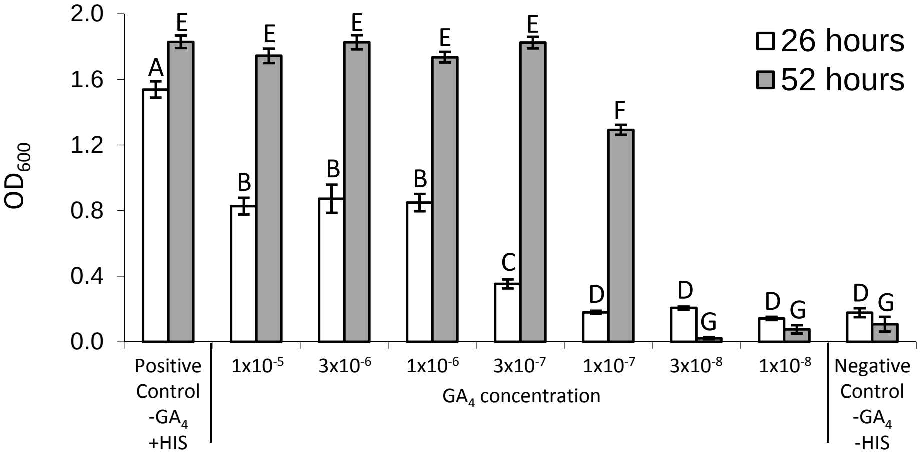

Supplementary Figure S12. GID1-DELLA yeast growth rates in response to different GA_4 concentrations. Optical density (OD_{600}) of yeast cultures supplemented with different levels of GA. Letters above bars represent pairwise statistical comparison between categories. Categories marked with a given letter are statistically different from categories marked with another letter (Student's *t*-test $p < 0.05$). All error bars are S.E.M.

Supplementary Figure S13. MEME motif patterns for each of the 516 2ODD genes examined.

Figure too large for PDF; See separate TIFF file
