# Supplementary figures and images for: Manipulating Gibberellin Control Over Growth and Fertility as a Possible Target for Managing Wild Radish Weed Populations in Cropping Systems
Source: Front Plant Sci. 2020 Mar 19;11:190. doi: 10.3389/fpls.2020.00190 (PMC7096587; doi:10.3389/fpls.2020.00190)

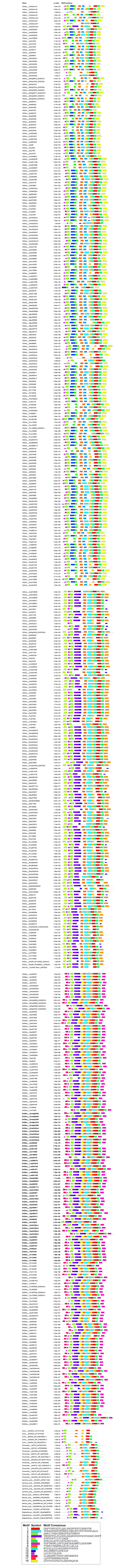

Supplement: Supplementary file 2 [file DataSheet_2.zip › Supplementary FIgure 13.TIF]

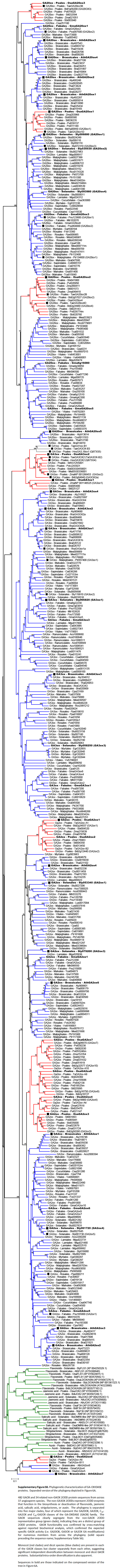

Supplement: Supplementary file 2 [file DataSheet_2.zip › Supplementary Figure 8.TIF]
